# A polymeric immunoglobulin—antigen fusion protein strategy for enhancing vaccine immunogenicity

**DOI:** 10.1111/pbi.12932

**Published:** 2018-07-21

**Authors:** Gina R. Webster, Craig van Dolleweerd, Thais Guerra, Szymon Stelter, Sven Hofmann, Mi‐Young Kim, Audrey Y‐H. Teh, Gil Reynolds Diogo, Alastair Copland, Mathew J. Paul, Peter Hart, Rajko Reljic, Julian K‐C. Ma

**Affiliations:** ^1^ Institute for Infection and Immunity St. George's University of London London UK; ^2^ University of Canterbury Christchurch New Zealand

**Keywords:** plant biotechnology, fusion protein, tuberculosis, Ag85B, immune complex, FcγR

## Abstract

In this study, a strategy based on polymeric immunoglobulin G scaffolds (PIGS) was used to produce a vaccine candidate for *Mycobacterium tuberculosis*. A genetic fusion construct comprising genes encoding the mycobacterial Ag85B antigen, an immunoglobulin γ‐chain fragment and the tailpiece from immunoglobulin μ chain was engineered. Expression was attempted in Chinese Hamster Ovary (CHO) cells and in *Nicotiana benthamiana*. The recombinant protein assembled into polymeric structures (TB‐PIGS) in *N. benthamiana*, similar in size to polymeric IgM. These complexes were subsequently shown to bind to the complement protein C1q and FcγRs with increased affinity. Modification of the N‐glycans linked to TB‐PIGS by removal of xylose and fucose residues that are normally found in plant glycosylated proteins also resulted in increased affinity for low‐affinity FcγRs. Immunization studies in mice indicated that TB‐PIGS are highly immunogenic with and without adjuvant. However, they did not improve protective efficacy in mice against challenge with *M. tuberculosis* compared to conventional vaccination with BCG, suggesting that additional or alternative antigens may be needed to protect against this disease. Nevertheless, these results establish a novel platform for producing polymeric antigen‐IgG γ‐chain molecules with inherent functional characteristics that are desirable in vaccines.

## Introduction

Vaccination against infectious disease has been one of the great medical success stories of the last century. However, there remain many diseases for which vaccines remain undeveloped, as well as diseases where better vaccines are urgently needed. Modern vaccine development also prioritizes strategies to improve accessibility, availability and acceptability, which include cost, mode of administration and administration regime.

Vaccines based on recombinant protein antigens are attractive for many reasons, but administration of antigen alone is rarely sufficient to stimulate a potent, long‐lasting immune response. In almost all cases, an adjuvant is required to boost immunogenicity or to favour a specific kind of immune response. Adjuvant development is a major challenge for the vaccine industry (Reed *et al*., 2013). For this reason, antigens that have ‘self‐adjuvanting’ properties are of interest, as they would greatly simplify vaccine manufacture and development.

In the early stages of a normal immune response, the formation of natural immune complexes is an important step for targeting antigen‐presenting cells (APCs), activation of the classical complement cascade and other immunoregulatory functions (Wen *et al*., 2016). An approach to produce recombinant monoclonal immune complexes has been described using plant biotechnology, which results in the desired Fc effector functionality and proof of concept for enhanced immunogenicity was described for tetanus toxin (Chargelegue *et al*., 2005), Ebola virus glycoprotein (Phoolcharoen *et al*., 2011), *Mycobacterium tuberculosis* Ag85B (Pepponi *et al*., 2014) and a dengue vaccine candidate (Kim *et al*., 2015).

An alternative approach to assembling and expressing recombinant immune complexes has recently been described, using a strategy involving a polymeric immunoglobulin G scaffold (Mekhaiel *et al*., 2011). Hexa‐Fc fusion proteins were engineered using a variety of proteins of interest, fused to the Fc region of human IgG1. The addition of the 18 amino acid tailpiece from human IgM resulted in well‐defined hexameric structures, which bound differently to complement, Fc receptors and B cells compared to their monomeric counterparts (Mekhaiel *et al*., 2011).

In this study, we investigate the use of the polymeric immunoglobulin G scaffold to create a multivalent vaccine candidate (TB‐PIGS) based on the Ag85B antigen of *M. tuberculosis*. Ag85B is the major component of the Ag85 complex, which in turn, is the major secreted component in mycobacterial culture fluids (Wiker and Harboe, 1992). Early promising results with Ag85B led to its inclusion in the vaccine candidate rBCG30 as well as being fused to a plethora of other proteins such as ESAT‐6 in subunit vaccine candidates (Horwitz *et al*., 1995, 2000), and it is still a priority candidate for current vaccine developers (Babaki *et al*., 2017).

We compare the expression of TB‐PIGS incorporating mouse IgG2a and human IgG1 sequences, and we also assess their expression in CHO cells and transient expression in *Nicotiana benthamiana*. We report characterization of the assembly of the TB‐PIGS, their size and *in vitro* functional properties. Finally, we describe the immunogenicity of TB‐PIGS in a mouse model, as well as the response to challenge with *M. tuberculosis*.

## Results

### Design and expression of TB‐PIGS in wild‐type and transgenic ΔXF plants

**Figure 1:**
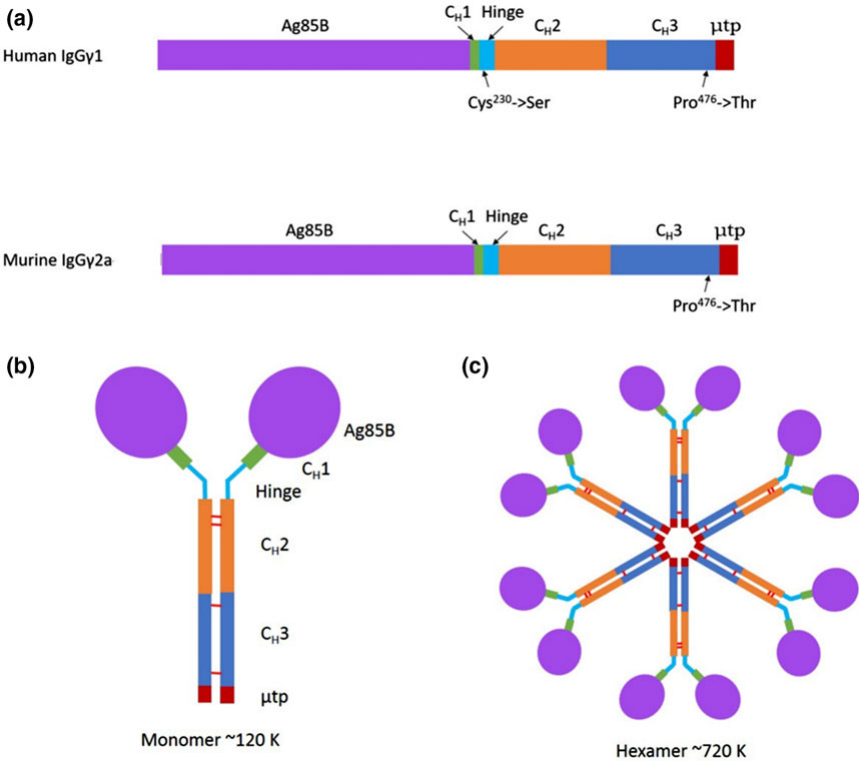
Design of TB‐PIGS. (a) Linearized map of gene sequence for TB‐PIGS. The main structural elements are indicated for both human and murine immunoglobulin‐based scaffolds with the amino acid substitutions highlighted. (b) Diagrammatic representation of TB‐PIGS ‘monomer’. Expected size of ~120 K, not including any glycosylation. Diagram is not to scale; Ag85B portion is ~30 K and truncated γ‐chain is ~30 K. (c) Diagrammatic representation of TB‐PIGS ‘polymer’. Expected size of ~720 K for hexamer, not including any glycosylation. There are potentially six glycosylation sites in one single chain: four within Ag85B nucleotide sequence and two within truncated γ‐chain. However, only two sites are predicted to be accessible for glycosylation to occur when protein is folded into 3D structure.

DNA sequences for murine (Mseγ_2a)_ and human (Huγ_1)_ heavy chain scaffolds were synthesized using commercial codon optimization algorithms for plant and mammalian expression. The scaffolds contained the nucleotide sequence encoding the C_H_2 and C_H_3 domains as well as the last 9–10 amino acid residues of the C_H_1 domain for either Mseγ_2a_ or Huγ_1_ (Figure 1). For Huγ_1_ constructs, Cys^230^ was substituted to Ser in the hinge region, to prevent disulphide bond formation occurring, in the absence of light chain. The Ag85B sequence was incorporated at the 5′end of each scaffold sequence.

The μtp from IgM was added to the C‐terminus of the CH_3_ domain of the γ‐chains, and in both mouse and human versions, Pro^476^ was substituted to Thr to ensure that the terminal residues of the C_H_3 domain of the γ‐chain are identical to those of the C_H_4 domain of the μ‐chain. This had previously been suggested to help integrate the two immunoglobulin domains together and prevent disruption of IgM polymeric structural abilities (Smith *et al*., 1995). IgM predominantly forms hexameric molecules in the absence of J‐chain and pentameric molecules in the presence of J‐chain (Randall *et al*., 1992). Assembly of TB‐PIGS into monomeric (IgG‐like) structures would result in molecular structures of approximate *M*_r_ 120 K. Subsequent hexameric assembly (as no J‐chain will be present) would result in molecular structures of approximate *M*_r_ 720 K (Figure 1).

Initially, expression was attempted in mammalian CHO cells which were transfected with pEF‐DEST51::TB‐PIGS vectors, using both murine‐ and human‐based scaffolds. The transfected CHO cells were selected using blasticidin. Although CHO cell transfections were attempted repeatedly, no successful transfectants were recovered for TB‐PIGS. Successful transfections were, however, recovered on each occasion for a control PIGS protein, comprising the immunoglobulin scaffold without antigen (FigureS1).

Two *N. benthamiana* plant lines were vacuum infiltrated with *Agrobacteria* containing pTRAk.2::TB‐PIGS vectors, using both murine‐ and human‐based scaffolds. Wild‐type *N. benthamiana* results in recombinant protein production with typical plant glycosylation, whereas a glyco‐engineered *N. benthamiana* line in which xylosyl‐ and fucosyl‐transferases were deleted, results in glycoproteins with glycans that are more similar to those commonly found in mammalian systems (Strasser *et al*., 2008). Five days postinfiltration, the leaves were harvested and soluble protein was extracted. Recombinant antibody molecules were purified by protein A affinity chromatography.

### Expression and assembly of TB‐PIGS

**Figure 2:**
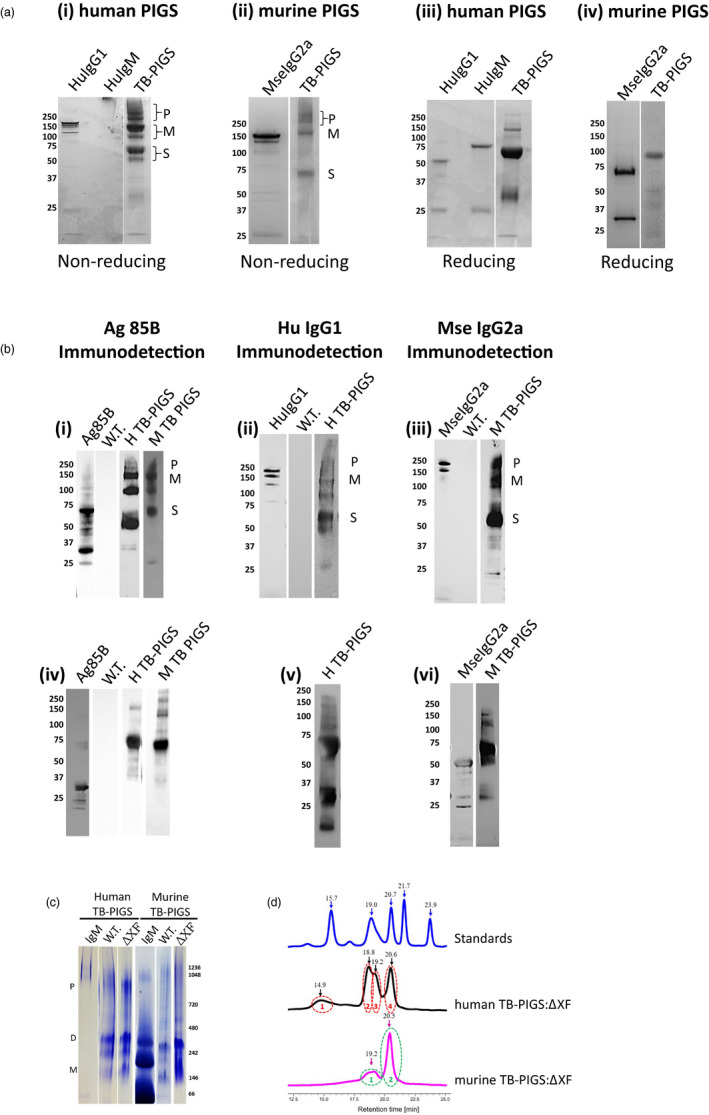
Characterization of assembly and size of TB‐PIGS. In all cases, 4%–12% Bis‐Tris gels with MOPS buffer were used. ~5–10 μg of protein was loaded per well. (a) SDS‐PAGE of purified human and murine TB‐PIGS detected by Coomassie Blue staining. Human IgG1, IgM and murine IgG2a are shown as controls. Panels i‐ii are under nonreducing conditions; panels iii‐iv are under reducing conditions. (b) Western blotting—Panels i and iv show detection using α‐Ag85B antiserum under nonreducing and reducing conditions, respectively. Positive control is plant expressed Ag85B; negative control is an extract from a wild‐type *Nicotiana benthamiana* plant (W.T.). Samples are purified human (H) and murine (M) TB‐PIGS; panels ii and v show detection using α‐human IgG1 antiserum under nonreducing and reducing conditions, respectively. Positive control is commercial purified human IgG1; panels iii and vi show detection using α‐murine IgG2a antiserum under nonreducing and reducing conditions, respectively. Positive control is commercial purified murine IgG2a; P indicates polymer; M indicates monomer; and S indicates single chain size of TB‐PIGS. Molecular weight markers in K are indicated to the left of each gel. (c) Native PAGE of TB‐PIGS. A 3%–12% bis‐tris gel in dark blue cathode and anode buffer was used. Molecular size markers are indicated on right‐hand side of gel. P ~ polymer size; D ~ dimer size and M ~ monomer size. (d) Size exclusion chromatography‐ultraviolet spectrophotometry (SEC‐UV). Bio‐Rad Standards include (from right to left) 1.35 kDa Vitamin B12 (0.5 mg), 17 kDa Horse Myoglobin (2.5 mg), 44 kDa Chicken Ovalbumin (5 mg), 158 kDa Bovine γ‐globulin (5 mg) and 670 kDa Bovine Thyroglobulin (5 mg). A total volume of 20 μL was run for each sample. Human TB‐PIGS:ΔXF was run at a sample concentration of 100 μg/mL and yielded four peaks. Murine TB‐PIGS:ΔXF was run at a concentration of 100 μg/mL and yielded two peaks. Black numbers indicate the retention time of each peak.

Plant samples were analysed by SDS‐PAGE and western blotting using antiserum specific for Ag85B, human γ1 or mouse γ2a immunoglobulin chains (Figure 2). Under nonreducing conditions, Coomassie Blue‐stained SDS‐PAGE demonstrates three major protein groups for both Human TB‐PIGS and Murine TB‐PIGS (Figure 2a panels i‐ii). There was a major band at approx. *M*_r_ ~120 K (labelled M), which is the predicted size for the PIGS monomer (Figure 1). Protein bands of higher *M*_r_ are also present (~250 K and greater, labelled P), which are likely to represent polymeric structures. There are also major bands at *M*_r_ ~60 K (labelled S) which may be single chain, unassembled fusion proteins or degradation products. For comparison, commercial purified human IgG1, human IgM and murine IgG2a were also analysed on the same gels. Under reducing conditions, the human TB‐PIGS sample resolved into a predominant band of approximate *M*_r_ ~60 K, which is likely to represent unassembled single fusion proteins (Figure 2a panels iii‐iv). A similar size band was observed for the murine TB‐PIGS, but the predominant band was *M*_r_ ~100 K, which may be a dimer. Some lower molecular bands were also evident, particularly in the case of the Human TB‐PIGS, which are likely to represent truncated degradation products. For comparison, reduced samples of commercial purified human IgG1, human IgM and murine IgG2a were also run, demonstrating bands at the expected *M*_r_ for immunoglobulin heavy and light chains.

The identity of the bands was supported by western blotting using antigen‐ and antibody‐specific antisera. Under nonreducing conditions, all the major protein bands in the human and murine TB‐PIGS samples were detected using both anti‐Ag85B and anti‐immunoglobulin heavy chain (anti‐Human γ1 or anti‐Mouse γ2a) antisera (Figure 2b panels i‐iii). Under reducing conditions, the major *M*_r_ ~60 K was detected with both anti‐Ag85B and anti‐immunoglobulin heavy chain antisera, but the lower *M*_r_ bands <37 K were only detected by the anti‐immunoglobulin heavy chain antisera (Figure 2b panels iv‐vi).

Similar results were obtained when the human and murine TB‐PIGS were expressed in glyco‐engineered (ΔXF) *N. benthamiana* (data not shown). Successful glyco‐engineering was confirmed by Western blotting using specific antisera to confirm the absence of xylose and fucose residues on recombinant proteins expressed in ΔXF plants (results not shown).

Fully assembled hexameric TB‐PIGS are very large proteins that would be difficult to visualize by SDS‐PAGE. Two different systems were therefore utilized to demonstrate polymeric assembly of TB‐PIGS more accurately: native PAGE (Figure 2c) and size exclusion chromatography‐ultraviolet spectrophotometry (SEC‐UV; Figure 2d).

Native PAGE (Figure 2c) indicated that human TB‐PIGS assembled into four major protein bands that were consistent with polymers (800–1000 K), dimers (two bands ~240–300 K) and probably monomers (~140 K). The murine TB‐PIGS, however, appeared predominantly as a single band (~250 K). Commercial human and murine IgM standards were run in parallel. Under SEC‐UV (Figure 2d), the murine TB‐PIGS (expressed in ΔXF plants) resolved into two peaks (retention time 19.2 and 20.5 mins), which are consistent with results expected for dimer/monomer and a single chain. Four species were observed in the human TB‐PIGS:ΔXF sample, with retention times consistent with those expected for polymers, dimers, monomers and single chain. The molecular weights of TB‐PIGS species were determined by comparison with retention times for proteins of known molecular weight within the Bio‐Rad standards sample [from left to right—Vitamin B12 (1.35 K), myoglobin (17 K), ovalbumin (44 K), γ‐globulin (158 K) and thyroglobulin (670 K)]. Yield estimations are provided in supplementary Table S1.

### Functional characterization of TB‐PIGS

**Figure 3:**
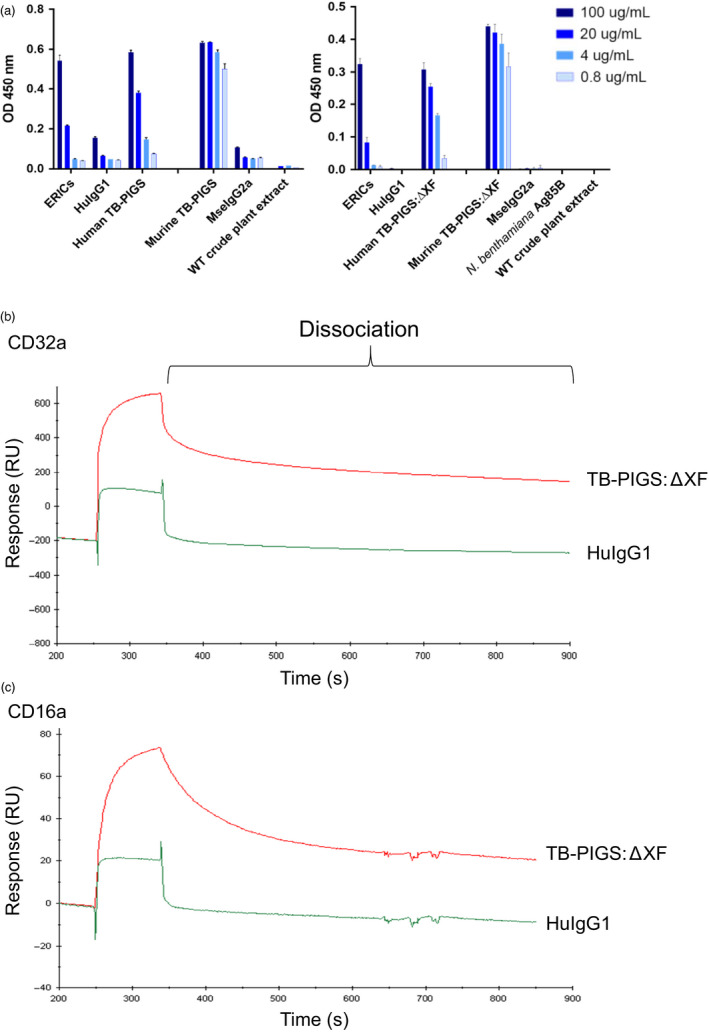
Functional characterization of TB‐PIGS *in vitro*. (a) Complement‐binding ELISA. ELISA plates were coated with 5 μg/mL human C1q. Positive control was ICMs (TBG65 and ACR). α‐Mse γ1‐HRP was used as detection antibody. Human TB‐PIGS and HuIgG1 control were detected using α‐Huγ_1_ antiserum. Murine TB‐PIGS and MseIgG2a control were detected using α‐Mseγ_2a_ antiserum. (b) SPR analysis of avidity interaction between glyco‐engineered human TB‐PIGS and human CD32a. 1 μM of human TB‐PIGS:ΔXF or HuIgG1 was passed over the His‐captured human CD32a. (c) Avidity analysis between glyco‐engineered human TB‐PIGS and human CD16a. 540 nM of human TB‐PIGS:ΔXF or HuIgG1 was passed over the His‐captured human CD16a.

To assess functionality of TB‐PIGS, their binding to C1q (the first component of the classical complement pathway) was determined by ELISA (Figure 3a). Both the human and murine TB‐PIGS, produced in wild‐type and glyco‐engineered ΔXF plants, bound to C1q in a concentration‐dependent manner. WT crude plant extract, Ag85B, MseIgG2a and HuIgG1 (all used as negative controls) only resulted in minimal or no binding to C1q. In contrast, positive control (consisting of murine IgG1 monoclonal antibody bound to polymeric *M. tuberculosis* antigen Acr in an immune complex) bound to C1q.

Surface plasmon resonance (SPR) was used to determine affinity and kinetics of binding between human TB‐PIGS and the high‐affinity receptor CD64 (FcγRI) or low‐affinity receptors CD16a and CD32a (FcγRIIIa and FcγRIIa) (FigureS2 and Table S2). This experiment was to determine the individual interaction of FcγR with a single ‘arm’ of the polymeric TB‐PIGS and to determine that the FcγR‐binding site on the engineered IgG‐like structure retains its proper activity. Human TB‐PIGS produced in ΔXF plants had high‐affinity binding to CD64 (0.41 nM) which was similar to the affinity of human IgG1 with the same receptor (0.27 nM) as expected. Human TB‐PIGS expressed with normal plant glycosylation had approximately 10‐fold lower affinity binding to CD64. The affinity of human TB‐PIGS to CD16a was superior to human IgG1 as expected. In particular, the K_D_ for glyco‐engineered human TB‐PIGS was 19.7 nM compared with the K_D_ for human IgG1 of 673 nM. The binding affinities of human IgG1 and human TB‐PIGS expressed in WT or ΔXF plants were similar and in the micromolar range.

Surface plasmon resonance was also used to demonstrate differences in avidity between glyco‐engineered human TB‐PIGS:ΔXF with low‐affinity FcγRs, compared to monomeric (commercial) HuIgG1. The experimental set‐up involved tethering multiple FcγRs to the chip surface allowing polymeric TB‐PIGS to bind to multiple receptors simultaneously, allowing avidity measurements. Although avidity cannot be quantified accurately in this assay, the differences between TB‐PIGS:ΔXF and HuIgG1 are evident from the dissociation curves (Figure 3b, c). For both CD32a and CD16a, the dissociation of HuIgG1 is rapid and almost complete within seconds. In contrast, the TB‐PIGS:ΔXF dissociate more slowly, particularly from CD32a.

### Binding of TB‐PIGS to monocyte cell lines

**Figure 4:**
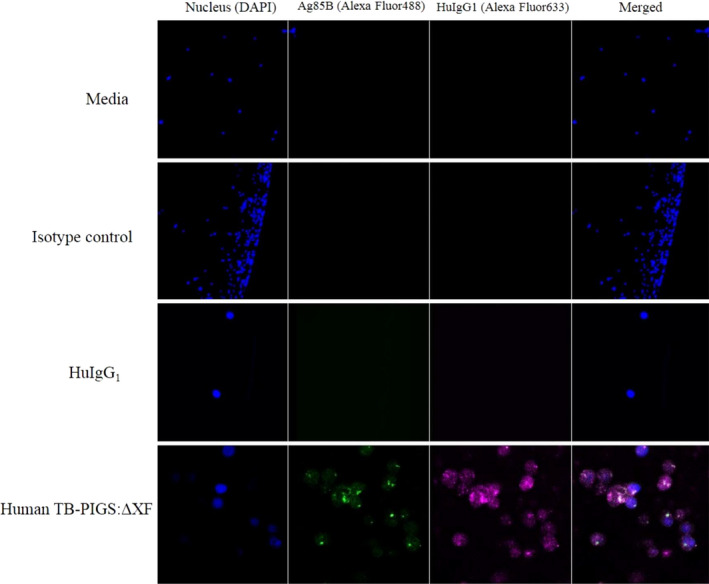
Confocal microscopy of THP1 cells with human TB‐PIGS:ΔXF. Nuclei were stained with DAPI (blue); Ag85B was detected with rabbit α‐Ag85B antiserum and goat α‐rabbit Ig‐Alexa Fluor 488 (green); HuIgG γ‐chain was detected with sheep α‐HuIgG1 antiserum and donkey α‐sheep IgG‐Alexa Fluor 633 (magenta). Superimposed images are shown (far right). In descending order, samples incubated with cells were; media only, isotype control (rabbit IgG‐Alexa Fluor 488), HuIgG1 and human TB‐PIGS:ΔXF.

Surface plasmon resonance demonstrated binding of TB‐PIGS to soluble FcγRs. This activity was next confirmed *ex vivo* by demonstrating binding to human TB‐PIGS by THP‐1 cells (a human monocyte cell line expressing FcγRs on their cellular surface) by both flow cytometry (Figure S3) and confocal microscopy (Figure 4).

THP‐1 cells were pretreated with commercial ‘Fc block’, to block binding to CD64, then incubated with either medium, a rabbit IgG antiserum or HuIgG1 as controls, and human TB‐PIGS produced in ΔXF plants as indicated. THP‐1 nuclear staining was performed with DAPI (blue—first column). Detection of antibody or TB‐PIGS binding to the cells was with either rabbit α‐Ag85B antiserum, followed by a goat α‐Rabbit Ig‐Alexa Fluor488 antiserum (green—second column), or sheep α‐HuIgG1 followed by donkey α‐sheep IgG‐Alexa Fluor633 (magenta—third column). Superimposed images are shown in the fourth column.

After blocking CD64, HuIgG1 did not bind to the low‐affinity FcγRs as expected (Figure 4 row 3). Human TB‐PIGS:ΔXF binding to THP‐1 was detected using both α‐Ag85B and α‐HuIgG1 antisera (Figure 4 row 4). This result was supported by the flow cytometry analysis, which demonstrated very low level of fluorescence for HuIgG1 (Figure S3), increased fluorescent signal for human TB‐PIGS:ΔXF, consistent with the signal for heat aggregated IgG1 which was used as the positive control. The frequency and distribution of polyfunctional T‐cells is shown in suppl. Figure S6.

Similar results were observed for binding of murine TB‐PIGS to FcγRs on the surface of J774 cells (a mouse monocyte/macrophage cell line), although in this case, high‐affinity receptors were not blocked, so binding to these receptors was observed using MseIgG2a control (Figure S5). Use of phalloidin and DAPI staining for cytoplasm and nuclei, respectively, indicated that TB‐PIGS bind to receptors on the surface of the cell and may be taken up by the cell (Figures S4).

### *In vivo* immunogenicity and protective efficacy elicited by TB‐PIGS

**Figure 5:**
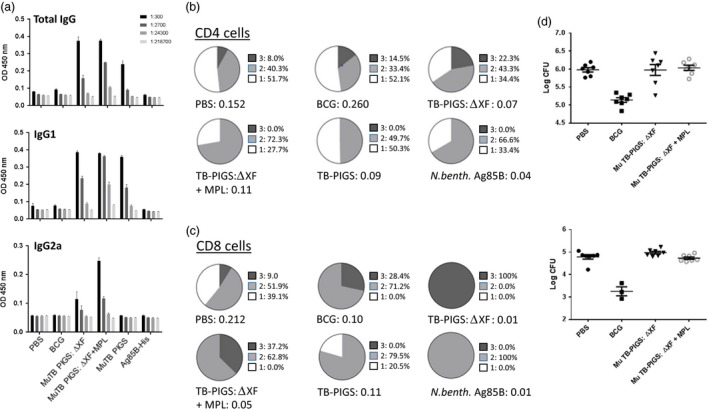
*In vivo* study of murine TB‐PIGS in BALB/c mice. (a) Ag85B‐specific IgG responses. Three weeks after last immunization, three mice were culled per group, and serum was obtained. ELISA plates were coated with Ag85B, and mean (±SEM) are depicted for total Ag85b‐specific IgG responses (top), IgG1‐specific response (middle) and IgG2a‐specific response (bottom). (b) Polyfunctional CD4^+^ T‐cell responses. Splenocytes were stimulated with Ag85B and stained for four different cytokines (IFN‐γ, IL‐2, IL‐17 and TNF‐α). Analysis of the percentage of positive CD4^+^ T cells for each cytokine alone and in combination was determined and represented in pie charts (top). The total percentage of cells out of the events counted (before gating) are listed under each pie charts. (c) Polyfunctional CD8^+^ T‐cell responses. As for (b) but for CD8^+^ cells. (d) *Mycobacterium tuberculosis* challenge and tissue colonization. Extracts of lungs (left) and spleens (right) were plated out at three different dilutions per mouse, incubated for 3 weeks and the number of mycobacterial colonies counted. Mean (±SEM) are plotted per group. A one‐way ANOVA with Tukey's multiple comparisons test was performed to determine statistical significance.

BALB/c mice were immunized with murine TB‐PIGS produced in both wild‐type and ΔXF *N. benthamiana*, with and without adjuvant. TB‐PIGS were highly immunogenic, but plant‐produced Ag85B alone was not (Figure 5 a–c). An Ag85B‐specific antibody response was observed in all animals immunized with Murine TB‐PIGS (Figure 5a). Murine TB‐PIGS alone in particular induced a strong IgG1 (Th2‐type) response, but this shifted to a mixed Th1/Th2 response with the addition of MPL adjuvant, as indicated by production of α‐Ag85B‐specific IgG2a antibodies.

Ag85B‐specific T‐cell proliferative responses were also observed following an *in vitro* recall assay involving stimulation of splenocyte cultures with Ag85B. Figure 5b, c show the analysis of specific polyfunctional CD4^+^ and CD8^+^ cells, respectively. Surprisingly, the PBS control group was found to have some specific CD4^+^ T‐cell response. However, TB‐PIGS from ΔXF *N. benthamiana* but not WT plants induced a higher proportion of triple‐positive cells compared to either PBS or BCG groups. Addition of MPL adjuvant increased the proportion of double‐positive cells but abolished the triple‐positive cells, similar to antigen stimulation alone. In the CD8^+^ T‐cell compartment, the striking finding was that TB‐PIGS from ΔXF *N. benthamiana* induced exclusively triple‐positive cells and was superior to all other groups, including when combined with the MPL adjuvant. Overall, the data showed that TB‐PIGS from ΔXF *N. benthamiana* are highly potent in inducing triple‐positive CD4^+^ and CD8^+^ T cells and that this was largely reversed by the addition of MPL adjuvant.

Finally, the potential protective efficacy of TB‐PIGS was assessed following intranasal aerosol challenge with *M. tuberculosis*. By assessment of recoverable bacterial counts from lungs and spleens, no improvement in protective efficacy compared to PBS immunized mice was observed (Figure 5d).

To test the human TB‐PIGS, a similar study was performed using transgenic CD64 (human FcγRI) mice. Immunization was with human TB‐PIGS, produced in ΔXF *N. benthamiana*, with and without BCG priming. Two adjuvants were tested: MPL and poly(I:C).

**Figure 6:**
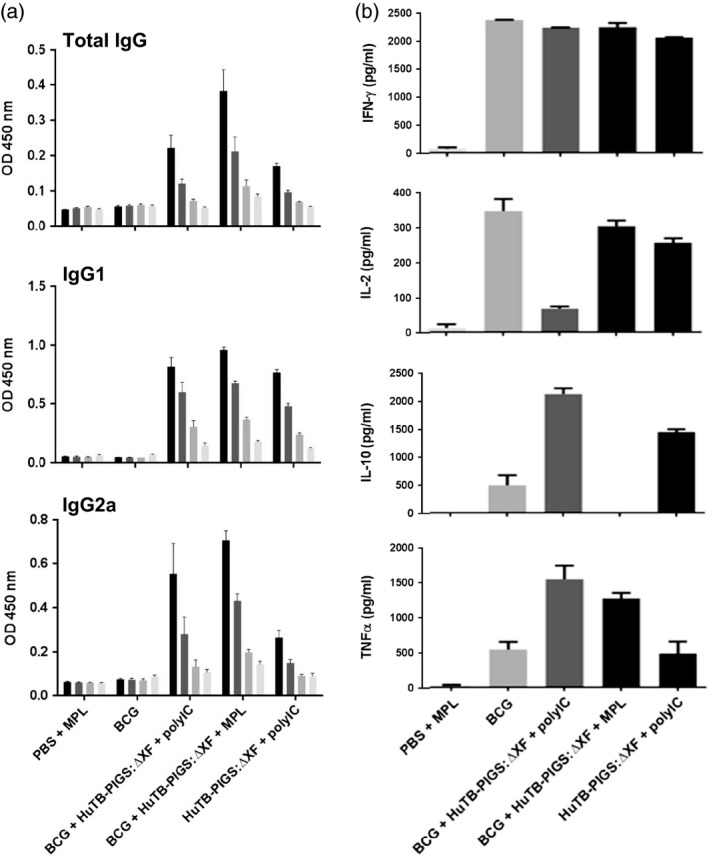
*In vivo* study of human TB‐PIGS in transgenic CD64 mice. (a) α‐Ag85B‐specific IgG responses. Three weeks after last immunization, three mice were culled per group and serum was obtained. ELISA plates were coated with Ag85B and mean (±SEM) are depicted for total IgG responses (top), IgG1‐specific response (middle) and IgG2a‐specific response (bottom). (b) Cytokine response. Splenocytes were stimulated with Ag85B, and levels of four different cytokines, in the supernatant, were determined by ELISA. A one‐way ANOVA with Tukey's multiple comparisons test was performed to determine statistical significance.

As with the Murine TB‐PIGS, an Ag85B‐specific antibody response was elicited in all animals immunized with Human TB‐PIGS (Figure 6a). Similar mixed IgG1/IgG2a responses were seen in the three groups, all of which received an adjuvant. BCG priming did not have a significant effect.

**Figure 7:**
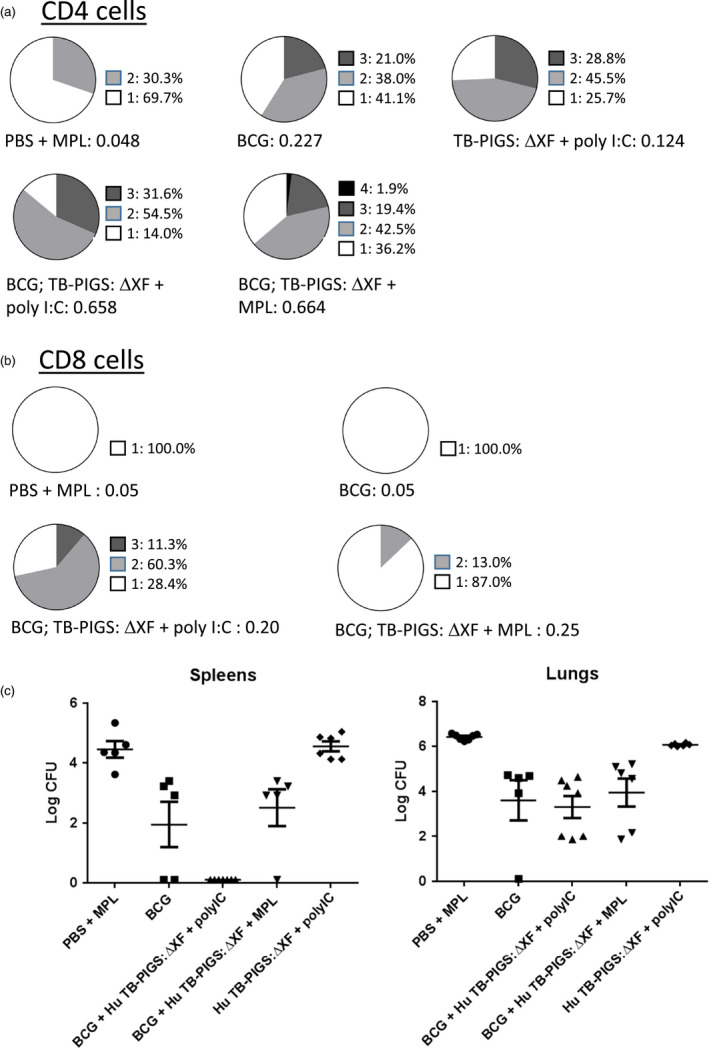
*In vivo* study of human TB‐PIGS in transgenic CD64 mice. (a) Polyfunctional CD4^+^ T‐cell responses. Splenocytes were stimulated with Ag85B and stained for four different cytokines (IFN‐γ, IL‐2, IL‐17 and TNF‐α). Analysis of the percentage of positive CD4^+^ T cells for each cytokine alone and in combination was determined and represented in pie charts (top). The total percentage of cells out of the events counted (before gating) are listed under the pie charts. (b) Polyfunctional CD8^+^ T‐cell responses. As for (a) but for CD8^+^ cells. (c) Protective efficacy of human TB‐PIGS against challenge with *Mycobacterium tuberculosis*. Extracts of lungs (left) and spleens (right) were plated out at three different dilutions per mouse and incubated for 3 weeks, and then, the number of mycobacterial colonies counted. Mean (±SEM) are plotted per group.

Analysis of splenocyte cytokine responses (Figure 6b) also suggests a mixed Th1/Th2 response. All groups except PBS + MPL control group resulted in high levels of IFN‐γ. If mice did not receive BCG, statistically lower levels of IL‐2 were produced. Poly(I:C), with or without BCG priming, resulted in increased levels of TNF‐α, compared to control mice or those receiving MPL adjuvant. However, the most balanced cytokine response appeared in the group that was primed with BCG and received Human TB‐PIGS:ΔXF + MPL adjuvant. Furthermore, analysis of polyfunctional T cells (Figure 7a) indicated that this was the only group in which CD4^+^ quadruple cytokine‐producing T cells were elicited. A high proportion of triple (IFN‐γ, IL‐2 and TNF‐α) and double (IFN‐γ and IL‐17) cytokine‐producing CD4^+^ cells was also observed in this group, as well as in the BCG primed group that received human TB‐PIGS:ΔXF with poly(I:C) adjuvant. CD8^+^ cells were largely restricted to single cytokine producers in all groups, except for this latter group that was primed with BCG and boosted with human TB‐PIGS:ΔXF + poly(I:C) adjuvant. Here, there was a significant proportion of double and triple cytokine‐producing T cells (Figure 7b). The frequency and distribution of polyfunctional T‐cells is shown in suppl. Figure S6. In both cases, mice primed with BCG and boosted with human TB‐PIGS resulted in greater numbers of α‐Ag85B‐specific T cells compared to control groups.

Finally, none of the vaccine candidate groups resulted in a statistical reduction in bacterial load in the lungs, compared to mice immunized with BCG (*p* < 0.0001) (Figure 7). However, mice primed with BCG and boosted with human TB‐PIGS:ΔXF + poly(I:C) adjuvant resulted in a statistically significant reduction in bacterial load in the spleens, compared to mice immunized with BCG (*p* < 0.0001) (Figure 7).

## Discussion

Tuberculosis is a major global health problem, due to limitations of the BCG vaccine, the rise of multidrug resistant and extensively drug‐resistant TB cases, as well as the increased prevalence of immunocompromised people (Cayabyab *et al*., 2012). A considerable effort is therefore being made to develop a new TB vaccine to either replace or boost BCG‐immunization. Ag85B is an established vaccine candidate and was used in this study as a model antigen to assess a novel molecular platform for vaccine design. PIGS is an attractive platform because it is designed to present multiple copies of antigen whilst also directly targeting APCs through Fc receptors to enhance antigen uptake and presentation.

Two expression systems were tested for production of the TB‐PIGS. CHO cells were selected because they are currently the industry standard for recombinant immunoglobulin pharmaceuticals and transient expression in *N. benthamiana,* an emerging plant expression system with significant potential advantages in relation to pharmaceuticals that are urgently needed in low‐ and middle‐income countries (Chen and Davis, 2016). Nicotiana plants are well documented to express antibodies and antibody complexes extremely efficiently and accurately (Chargelegue *et al*., 2005; Hiatt *et al*., 1989; Ma *et al*., 1995; Teh *et al*., 2014). Unexpectedly, we were unable to express any TB‐PIGS in CHO cells. The PIG scaffold alone, lacking Ag85B, was expressed successfully in CHO cells, suggesting a protein‐specific issue, such as toxicity of the antigen or the overall size of TB‐PIGS.

We generated a murine γ2a scaffold first because we planned to perform pilot *in vivo* testing in mice, but we also designed and expressed a human γ1 scaffold in anticipation of developing a vaccine product. Both could be expressed in *N. benthamiana* although differences in polymerization states were observed. Native PAGE indicated that the human scaffold resulted in a greater proportion of polymers (~35%–60%) compared to monomers (~1%–10%), whereas Mseγ_2a_ scaffolds resulted in predominantly monomers (~45%–60%) compared to polymers (~5%–20%). This finding was partly corroborated by SEC‐UV spectrophotometry. Mekhaiel *et al*. also found that Human IgG1‐based hexa‐Fc constructs could form hexameric structures, yet their Mouse IgG2a‐based constructs did not form anything larger than a dimer (Mekhaiel *et al*., 2011). The reason for these differences is unknown. Further improvement in polymer yields may require additional mutations, such as the L309C mutation in human IgG1 heavy chain, which has been indicated to increase yields of polymers, as it is homologous to Cys414 in IgM, which has been shown to have a role in interheavy chain disulphide bonds (Mekhaiel *et al*., 2011; Sorensen *et al*., 1999; Yoo *et al*., 1999). However, this mutation does not always improve polymer yields (Teye *et al*., 2017).

The PIG scaffold is designed to mimic polymeric IgM and the Fc‐mediated properties of that molecule. Functional assessment of the TB‐PIGS included assays to demonstrate binding to complement and Fcγ receptors. The classical complement pathway is normally activated by polymeric antigen‐bound IgG or IgM binding to C1q, leading to activation of the enzyme cascade. IgG normally exists in a monomeric state, in which the C1q binding site is accessible, but the affinity is too low to bind and activate C1q (Diebolder *et al*., 2014). IgG needs to polymerize to bind C1q with high avidity (Diebolder *et al*., 2014). The demonstration that TB‐PIGS could bind to C1q was therefore an indication of their polymeric status. These results support similar findings by Smith *et al*. but contrast with Mekhaiel *et al*., who showed that hexa‐Fc fusion molecules bound C1q poorly compared to dimers and monomers (Mekhaiel *et al*., 2011; Smith and Morrison, 1994; Smith *et al*., 1995). Mekhaiel *et al*. also showed that the interaction of their hexa‐Fc molecules with FcγRs was dependent on the fusion partner. They hypothesized that these results may be due to steric hindrance, caused by the antigen blocking the C1q or FcγR binding site located in or near the hinge region. Therefore, in our constructs, we included the last 9–10 amino acid residues of the C_H_1 domain, corresponding to a β‐strand, to add molecular flexibility and to avoid any potential steric interference.

The only known difference between antibodies expressed in plants and those expressed by mammalian cells is in N‐glycosylation. Although the initial steps of N‐glycosylation and N‐glycan processing are highly conserved between plants and mammals, subsequent N‐glycan maturation differs in plants resulting in complex N‐glycans with β1,2‐linked xylose and core alpha 1,3‐linked fucose that are not found in mammalian antibody glycosylation (Cabanes‐Macheteau *et al*., 1999; Strasser, 2016). Glycosylation is known to impact molecular interactions of IgG (Hayes *et al*., 2014; Hodoniczky *et al*., 2005; Shields *et al*., 2002; Zhou *et al*., 2008). In particular, *N*‐linked glycan structures present at Asn 297 in IgG can modulate FcγR binding, which affects the immune response induced (Mimura *et al*., 2001). For example, removal of fucose has been shown to improve binding of antibody to FcγRIIIa and results in enhanced antibody‐dependent cell‐mediated cytotoxicity (ADCC) (Ferrara *et al*., 2011; Shields *et al*., 2001), so here we made use of a glyco‐engineered line of *N. benthamiana* (Strasser *et al*., 2008) in which the endogenous α1,3‐fucosyltransferase was silenced. Our results showed that the use of the glyco‐engineered *N. benthamiana* host had no impact on expression or assembly of TB‐PIGS. There was no functional loss associated with TB‐PIGS expressed in glyco‐engineered plants; for example, they bound equally well to C1q, but there was an important enhancement of binding affinity to CD64 and CD16a as predicted from previous reports (Hiatt *et al*., 2014).

Physiologically, IgG binds with high affinity to FcγRI/CD64, whereas immune complexes bind preferentially to low‐affinity receptors (FcγRII/CD32 and FcγRIII/CD16) (Klaassen *et al*., 1988; Lux *et al*., 2013; White *et al*., 2001). In our studies, the human TB‐PIGS bound with high affinity to FcγRI/CD64 and the glyco‐engineered human TB‐PIGS bound with enhanced affinity to FcγRII/CD32a and FcγRIII/CD16a. Potentially, this indicates that like naturally formed immune complexes, human TB‐PIGS could interact efficiently with immature dendritic cells (DCs) (reviewed in Platzer *et al*., 2014), resulting in endocytosis and leading to maturation and migration of the DCs to secondary lymphoid organs, where cytokines are produced that activate specific naïve T cells. *M. tuberculosis* has evolved to evade the human immune system, for example, by ‘hiding’ inside macrophages. This indicates that a vaccine candidate should elicit T‐cell responses (reviewed in Scriba *et al*., 2017). Recently, B cells and antibodies have also been indicated to be important in controlling *M. tuberculosis* disease (reviewed in du Plessis *et al*., 2016). Moreover, phagocytosis and ADCC may be impaired during active tuberculosis disease, possibly due to a decrease in antibody avidity (Arias‐bouda *et al*., 2003; Perley *et al*., 2014) and/or down‐regulation of Fc receptor expression (Jacobsen *et al*., 2007; Laux da Costa *et al*., 2015; Maertzdorf *et al*., 2011a,b). Therefore, improvement of antibody avidity and Fc receptor engagement may be a crucial area of research for protection against *M. tuberculosis*.

Mouse TB‐PIGS affinities for mouse FcγRs were inconsistent, but generally lower than observed for human TB‐PIGS against human FcγRs (data not shown). These differences may be due to less efficient polymerization of the PIG scaffold. In addition, murine FcγR has been less well studied than their human counterpart, so there may be unrecognized biological differences. The classification of murine FcγR homologs is based on amino acid similarity, rather than ligand binding or cellular expression (Bruhns, 2015); for example, mouse FcγRIV has been suggested to be the functional equivalent of both human FcγRI and human FcγRIIIa (Mechetina *et al*., 2002; Nimmerjahn *et al*., 2005). Investigation of half‐lives for murine TB‐PIGS with mouse FcγRI was inconclusive, but suggested that the dissociation is much faster than for human reagents (data not shown), which is a consideration in the analysis of *in vivo* results.

*In vivo* murine and human TB‐PIGS were tested in conventional and human CD64 transgenic BALB/c mice, respectively. Murine TB‐PIGS (produced in wild‐type and ΔXF plants) generated humoral and cellular immunity with and without adjuvant, but failed to reduce the bacterial load in challenged mice, compared to BCG immunized mice. Murine TB‐PIGS:ΔXF did, however, result in a greater percentage of triple cytokine‐producing CD4^+^ and CD8^+^ cells, which have been suggested to be important in protecting against *M. tuberculosis* infection. In the second *in vivo* study, human TB‐PIGS were used to immunize transgenic CD64 mice with and without BCG priming. The best IgG2a‐specific response with quadruple cytokine‐producing CD4^+^ cells was generated by mice primed with BCG and boosted with human TB‐PIGS:ΔXF + MPL adjuvant. In contrast, the greatest proportion of triple cytokine‐producing CD8^+^ cells was found for mice immunized with BCG and boosted with human TB‐PIGS:ΔXF + poly(I:C). CFU counts indicated this was the most promising vaccine candidate as there was a statistically significant reduction in bacterial burden in spleens of challenged mice compared to BCG immunized mice, suggesting that this vaccine candidate could impair MTB dissemination *in vivo*. However, only a marginal reduction was observed in lungs of challenged mice, which is important as the lungs are the primary site of entry for the pathogen.

Tuberculosis poses some fundamental challenges in the evaluation of vaccine candidates. There are questions about the predictive value of animal models for human disease, and there is a lack of correlates of vaccine‐induced protection (Karp *et al*., 2015). In addition to testing humoral and cellular immunity, we tested protection against mycobacterial challenge, but this is a demanding end‐point for *in vivo* assessment of TB‐PIGS as a vaccine candidate for a number of reasons. Importantly, Ag85B, although extensively studied, has not yet been demonstrated to provide consistent protection against infection. So its role in this study was as a model immunogen, rather than as a confirmed vaccine protein. Secondly, one of the main attractions of the PIG scaffold is its potential for targeting Fcγ receptors on APCs. As noted above, the biology of murine Fcγ receptors in relation to their human counterparts still needs clarification, and as our results suggested less polymerization for murine TB‐PIGS and diminished binding affinity of murine TB‐PIGS to murine FcRs compared with human TB‐PIGS to human FcRs, the murine model may not be optimal for protection studies. Furthermore, although we were able to test the human TB‐PIGS in human CD64 transgenic mice, the animals were not transgenic for low‐affinity FcγRs such as CD16a, which are known to play an important role in ADCC (Gupta *et al*., 2005).

In this study, we have demonstrated the feasibility of polymeric IgG scaffolds as a vaccine platform. We illustrated expression, assembly and purification from *N. benthamiana,* and demonstrated *in vitro* and *in vivo* functional characteristics based on our hypothesis that PIGS would promote enhanced antigen presentation to the immune system. It builds on a parallel project using a dengue vaccine candidate (Kim *et al*., 2017, 2018) and demonstrates the generic potential of this platform to be used for vaccine candidates against a range of infectious diseases. Further work is now needed to optimize the formation of polymers, to enhance their stability and to maximize yields in the plant expression system in order to advance to safety and immunization studies in humans.

## Experimental procedures

### Construction of TB‐PIGS vectors

GeneArt (ThermoFisher) synthesized four codon optimized constructs in pMA‐T vector. Two genes were codon optimized for *Nicotiana tabacum* (human PIGS and murine PIGS) and the other two for *Homo sapiens* (human PIGS^H^ and murine PIGS^H^). The PIGS sequence included leader sequence, truncated γ‐chain (derived from either human IgGγ_1_ or mouse IgGγ_2a_) and μtp from IgM, all flanked by NcoI and XbaI restriction sites as well as attB gateway cloning sites.

Primer pairs containing BsaI restriction sites: 5′‐cagagcggtctcgatgcgaggtgttctctcgtcctggtttgcct‐3′ and 5′‐tgttcaggtctcaaagcacctgctcccaaagaagattgaag‐3′ were designed to amplify *N‐*terminally truncated Ag85B (signal peptide removed) DNA fragment from pGEM::Ag85B‐ESAT6 construct (Dedieu *et al*., 2010) into pMA‐T plasmid containing PIGS sequence by PCR to create pMA‐T::human TB‐PIGS and pMA‐T::murine TB‐PIGS.

AttB1 and attB2 gateway cloning sites were used to clone Ag85B‐human PIGS and Ag85B‐murine PIGS into pDONR‐Zeo, using the BP Clonase II enzyme protocol (Invitrogen). The flanking NcoI and XbaI sites were used to clone the desired genes into pTRAk.2 (plant expression destination vector; van Dolleweerd *et al*., 2014; Sack *et al*., 2007) using T4 DNA Ligase (NEB).

### Expression of TB‐PIGS and Ag85B in plants

*Escherichia coli* DH5α cells (Invitrogen) were transformed to amplify the plasmid, prior to electroporation of *Agrobacterium tumefaciens* (strain GV3101::pMP90RK). A 400 mL *A. tumefaciens* culture was grown, centrifuged at 3800 ***g*** for 20 min, and the pellet re‐suspended in plant infiltration buffer (10 mM MES, 10 mM MgCl_2_ and 100 μg/mL acetosyringone at pH 5.6) to an OD_600_ of 0.25. 6‐ to 8‐week‐old *N. benthamiana* plants were immersed in *A. tumefaciens* solution inside a vacuum chamber with a vacuum pressure of <100 mbar for ~2 min. After infiltration, the plants were maintained at 26 °C for ~5–6 days prior to leaf harvest for recombinant protein analysis.

### Purification and characterization of TB‐PIGS from plants

Whole leaves were frozen in liquid nitrogen, and PBS (pH 7.0) was used as extraction buffer. A blender was used to homogenize the leaf material and then passed through miracloth (Merck) prior to centrifugation at 17 000*g* for 30 min. The supernatant was syringed through a 0.22 μm filter before being purified on protein A‐sepharose (SIGMA), except Ag85B which was purified on a nickel (II) affinity column.

Eluted fractions were pooled, concentrated using Amicon Ultra‐4 centrifugal filter devices and dialysed to exchange buffer for PBS (pH 7.0). The integrity of the proteins was determined by SDS‐PAGE on NuPAGE 4%–12% Bis‐Tris gels (Novex), both nonreducing and reducing (10% β‐mercaptoethanol), and the gels were stained with Coomassie Instant Blue stain (Expedeon). Additionally, NativePAGE 3%–12% Bis‐Tris gels (Novex) were utilized with NativeMark Unstained Protein Standard as per manufacturer's protocol. ImageJ software was used to analyse the intensity of bands within each lane of the gel. A chromatogram was plotted and integration of the area under the curve used to determine a value for the per cent of each species present.

Proteins were also separated on a high‐performance SEC Yarra 300 × 7.8 column utilized on an HPLC LC2010A machine with PBS (pH 7.0) as buffer. Eluted fractions were compared against known high molecular weight standards (Bio‐Rad).

### C1q binding ELISA

Microtiter wells were coated with 5 μg/mL of human C1q (Merck Calbiochem) in PBS (pH 7.0) and incubated at room temperature for 2 h prior to blocking with 5% skimmed milk for 2 h. The purified proteins, in PBS, were added to wells at a starting concentration of 100 μg/mL and in fivefold dilutions in blocking buffer. Plates were incubated at room temperature for 2 h before being washed with dH_2_O + 0.1% Tween‐20. Plates were incubated with detection antiserum (α‐Hu IgGγ_1_‐HRP, α‐Mse IgGγ_1_‐HRP or α‐Mse IgGγ_2a_‐HRP), in blocking buffer, for 2 h. Finally, plates were washed with dH_2_O + 0.1% Tween‐20 before being developed with 50 μL of HRP substrate in buffer (Sigmafast OPD tablet and Sigmafast buffer with urea H_2_O_2_ tablet in 20 mL dH_2_O). Absorbance was measured at 450 nm (Tecan Infinite F200 PRO).

### Surface plasmon resonance

Surface plasmon resonance experiments were performed using the Biacore X100 instrument (GE Healthcare). Filter‐sterilized HBS‐EP^+^ buffer was used as running buffer and dilution buffer. For determining affinity values, flow cells of CM5 sensor chips were coupled with recombinant protein A (SIGMA) using amine‐coupled chemistry as described by the manufacturer. TB‐PIGS were captured onto the surface of the chip. For analyte HuCD64 or MseCD64 (R&D systems), a flow rate of 40 μL/min, a contact time of 135 s, dissociation time of 600 s and regeneration buffer of 100 mM glycine (pH 1.5) were used. For analyte HuCD32a and HuCD16a (R&D systems), a flow rate of 40 μL/min, a contact time of 90 s, dissociation time of 300 s and regeneration buffer of 100 mM glycine (pH 1.5) were used. In all experiments, data were zero adjusted and evaluated using Biacore ×100 software. For determining avidity, a His capture kit (GE Healthcare) was used to bind antihistidine antibody to the surface of a CM5 chip using amine‐coupled chemistry. HuCD32a or HuCD16a was captured onto the surface of the chip. For analyte TB‐PIGS, a flow rate of 35 μL/min, a contact time of 90 s, a dissociation time of 300 s and His capture kit regeneration buffer were used.

### Confocal microscopy

If human FcR block (BD Pharmingen) was utilized, cells were incubated with 2.5 μg per 1 × 10^6^ cells for 20 min at room temperature before addition of 25 μg of PIGS or control protein (HuIgG1 or MseIgG2a). Cells were incubated at room temperature for 2 h before supernatant was discarded. Cells were washed with PBS before addition of 150 μL blocking buffer (PBS + 2% FBS + 1% BSA + 0.2% Triton X‐100 + 0.05% Tween‐20) per well. The plate was incubated at room temperature for 1 h before washing with PBS and fixing cells with 4% paraformaldehyde (SIGMA) for 20 min. Cells were washed with PBS + 7% glycine and permeabilized with 0.1% Triton X‐100 for 5 min. Again, cells were washed with PBS before addition of primary antibodies [sheep α‐HuIgG (TBS), sheep α‐MseIgG (TBS), rabbit α‐Ag85B (Abcam) or phalloidin‐Alexa488 (LifeTechnologies)] in blocking buffer. Plates were incubated for 1 h at room temperature, in the dark. Cells were washed with PBS, and then, secondary antibodies (donkey α‐sheep IgG‐AlexaFluor633 (Life Technologies) or goat α‐rabbit IgG‐AlexaFluor488 (Abcam)), in blocking buffer, were added for 1 h, in the dark. Finally, cells were washed with IF wash (PBS + 0.2% Triton X‐100 + 0.05% Tween‐20), before DAPI was added for 15 s, washed twice more with IF wash and then addition of 200 μL PBS and stored at 4 °C in the dark until imaging using a Zeiss Confocal LSM 510 and analysis using ImageJ software.

### *In vivo* immunization of mice

All animals were housed in the Biological Research Facilities (BRF) at St. George's, University of London, under Home Office regulations. Six‐ to eight‐week‐old female BALB/c mice purchased from Charles River/Harlan were used in all experiments with murine TB‐PIGS and were maintained under specific pathogen‐free conditions. Previously determined positive human CD64 mice were used to breed offspring and tail‐bleeds were screened by PCR to identify positive human CD64 transgenic mice. Infected animals were housed in cages contained within purpose‐built plastic isolators under negative pressure, in a containment level 3 facility.

Mice were immunized with TB‐PIGS at 25 μg/dose administered subcutaneously (s.c.) or intranasally (i.n.) at 3‐week intervals on three separate occasions, either alone or suspended with 20% v/v MPL (SIGMA) or poly(I:C) adjuvant (Invivo Gen). BCG priming was performed 9 weeks before first boost, by immunizing mice with 2x10^6^ CFU/dose (BCG‐Pasteur).

Three mice per vaccine group were culled 3–4 weeks after final immunization. Mice were anaesthetized under isoflurane and cardiac puncture performed to collect blood. Spleens were removed into 5 mL R10 media. Blood was placed into a 37 °C incubator for 30 min, then at 4 °C for 2 h, centrifuged at 14,000 ***g*** for 15 min and serum collected and stored at −20 °C. Spleens for each group were combined and macerated through a 70‐μm cell strainer using a sterile syringe plunger and washed into a Petri dish with 25 mL of R10 media. Cells were centrifuged at 400*g* for 10 min and re‐suspended in 5 mL/spleen ACK red blood cell lysis buffer (ThermoFisher Scientific) for 7 min. 40 mL of R10 media was added to halt the reaction; cells were centrifuged for 10 min at 400*g*, washed with 20 mL R10 buffer, centrifuged 10 min at 400*g* and pellet re‐suspended in 10 mL R10 buffer. Viable cells were counted using 0.4% trypan blue dye, counting slides and Bio‐Rad TC20 automated cell counter.

### Humoral responses

Microtiter wells were coated for 2 h at 37 °C with 10 μg/mL of *N. benthamiana* produced Ag85B protein in PBS (pH 7.0). Plates were washed with dH_2_O before being blocked for 1 h, at 37 °C, with PBS + 5% BSA. Plates were washed with dH_2_O. The mouse serum was added to wells (1:100) and a threefold serial dilution in blocking buffer. Plates were incubated at 37 °C for 2 h before being washed again with dH_2_O. Plates were incubated with secondary antibody (α‐Mse IgG‐HRP (TBS), α‐Mse γ_1_‐HRP (Abcam) or α‐Mse γ_2a_‐HRP (STAR)), in blocking buffer, for 1 h at 37 °C. Finally, plates were washed with dH_2_O before being developed with 50 μL of HRP substrate in buffer (Sigmafast OPD tablet and Sigmafast buffer with urea H_2_O_2_ tablet in 20 mL dH_2_O) and the reaction stopped with 25 μL 2M H_2_SO_4_. Absorbance was measured at 450 nm (Tecan infinite F200 PRO).

### Cytokine responses

Splenocytes were seeded at a concentration of 5 × 10^5^/mL per well in a 96‐well U‐bottomed plate (Corning). Cells were stimulated with 10 μg/mL of *N. benthamiana* produced Ag85B or 5 μg/mL concanavalin A (ConA; SIGMA) as a positive control or R10 media as a negative control. Plates were incubated at 37 °C, 5% CO_2_ and 90% humidity for 72 h, and supernatants were harvested for cytokine ELISAs. Cytokine ELISAs were performed as per manufacturer's instructions (eBiosciences). eBiosciences Mouse Th1/Th2 ELISA Ready‐SET‐Go! kit detected IFN‐γ, IL‐2 and IL10. Mouse TNFα ELISA Ready‐SET‐Go! kit detected TNFα.

### Polyfunctional T‐cell analysis

Splenocytes were seeded at a concentration of 1.5 × 10^6^/mL per well in a 96‐well V‐bottomed plate. Cells were stimulated with R10 media containing 100 μg/mL of Brefeldin A (SIGMA) and 5 μg/mL of *N. benthamiana* produced Ag85B or 100 μg/mL Brefeldin A, 4 μg/mL Phorbol Myristate Acetate (PMA; SIGMA) and 20 μg/mL ionomycin calcium salt (SIGMA) as a positive control or 100 μg/mL Brefeldin A as a negative control. Plates were incubated at 37 °C, 5% CO_2_ and 90% humidity for 4 h. Plates were centrifuged at 400*g* for 5 min at 4 °C, and supernatant discarded. All cells (except two wells which were left as negative controls) were stained with viability dye eFluor 780 (1:2000; eBioscience) in PBS for 30 min at 4 °C, in the dark. Plates were washed with 200 μL PBS, centrifuged at 400*g* for 5 min, and supernatant discarded. 100 μL Cytofix (BD) was added to each cell pellet and incubated at 4 °C for 30 min, in the dark. The plates were washed with 200 μL FACS buffer, centrifuged at 400*g* for 5 min, and supernatants discarded. Pellets were re‐suspended in 200 μL FACS buffer and stored at 4 °C, in the dark, overnight.

The plates were centrifuged at 400*g* for 5 min, and supernatants were discarded. 200 μL Permeabilization Buffer (PBS + 0.5% BSA + 0.1% sodium azide + 0.5% saponin) was added to each well, centrifuged at 400*g* for 5 min, and supernatant discarded. 25 μL of staining cocktail (eFluor 780, α‐TNF‐α‐APC, α‐IL2‐PE, α‐IL17‐Cy7, α‐IFN‐γ‐PE‐Dazzle, α‐CD3‐FITC, α‐CD4‐PerCP‐Cy5.5 and α‐CD8‐AlexaFluor700) was added to each pellet and incubated for 45 min at 4 °C, in the dark. Fluorescence Minus One (FMO) stains were prepared as necessary and added to appropriate wells for 45 min at 4 °C, in the dark. Similarly, Ultra compensation beads (eBiosciences) were stained for 45 min at 4 °C in the dark and used for compensation. Cells were washed in 200 μL Permeabilization buffer, centrifuged at 400*g* for 5 min, and supernatants discarded. Cells were washed in 200 μL FACS buffer, centrifuged at 400*g* for 5 min, and supernatants discarded. Finally, cells were re‐suspended in 200 μL FACS buffer and stored at 4 °C until acquisition on the flow cytometer (LSRII; BD). Analysis performed using FlowJo version 10.

### *Mycobacterium tuberculosis* challenge

Mice were transferred to Category III facilities and infected with aerosolized 200–500 CFU *M. tuberculosis* H37Rv for 10 min using a Biaera machine with Aero MP software (Management Platform) with nose‐only chambers. They were culled 3 weeks *post*challenge. Lungs and spleens were removed and homogenized in 5 mL of dH_2_O + 0.1% Triton X‐100 using a Seward 80 stomacher and serially diluted in dH_2_O + 0.1% Triton X‐100 (1:5, 1:25 and 1:125 for lungs and 1:5, 1:25 and 1:75 for spleens), 30 μL of each dilution was plated in duplicate onto 7H11 agar plates. CFUs were counted 3 weeks later. Due to limits of detection, a log 2.2 value was attributed to spleen plates where no colonies were observed. Similarly, log 1.9 was attributed to lung plates where no colonies were observed.

### Additional methods

Cloning of Ag85B as control protein for expression in *N. benthamiana,* Gateway cloning of TB‐PIGS into mammalian expression vector, Western blotting to determine structure of PIGS, expression and detection of PIGS in CHO cells, flow cytometry methodology and maintenance of cell lines are described in supplementary material.

## Author contributions

Gina R. Webster designed and performed research, analysed data and wrote the manuscript. Craig van Dolleweerd and Audrey Teh designed research and analysed data. Thais Guerra & Mi‐young Kim aided with research. Szymon Stelter designed and aided SPR experiments. Sven Hofmann designed and aided SEC experiments. Gil Reynolds Diogo performed cardiac punctures, category III laboratory work and aided with animal experiments. Mathew Paul performed category III laboratory work and aided with animal experiments. Alastair Copland designed the polyfunctional assay and performed category III laboratory work. Peter Hart performed category III laboratory work. Rajko Reljic aided in design and analysis of animal work and performed category III laboratory work. Julian K‐C. Ma designed and supervised research, analysed data and wrote the manuscript

## Competing financial interests

The authors declare no competing financial interests.

## Supporting information


**Figure S1** SDS‐PAGE of purified murine and human PIGS (without antigen) produced from CHO cells, under non‐reducing and reducing conditions.

**Figure S2** SPR kinetics and affinity analysis of TB‐PIGS binding to FcγRs.

**Figure S3** Flow cytometric analysis of human TB‐PIGS:ΔXF with THP1 cells.

**Figure S4** Confocal microscopy of J774 cells with murine TB‐PIGS:ΔXF.

**Figure S5** Flow cytometric analysis of murine TB‐PIGS:ΔXF with J774 cells.

**Figure S6** Frequency of antigen‐specific cytokine producing cells after immunisation in transgenic CD64 mice.

**Table S1** Yields for TB‐PIGS produced in plants in mg of protein per kg of leaf mass.
**Table S2** Kinetic and Affinity values of TB‐PIGS with Fc?Rs.
