# Supplementary figures and images for: A polymeric immunoglobulin—antigen fusion protein strategy for enhancing vaccine immunogenicity
Source: Plant Biotechnol J. 2018 Jul 21;16(12):1983–96. doi: 10.1111/pbi.12932 (PMC6230950; doi:10.1111/pbi.12932)

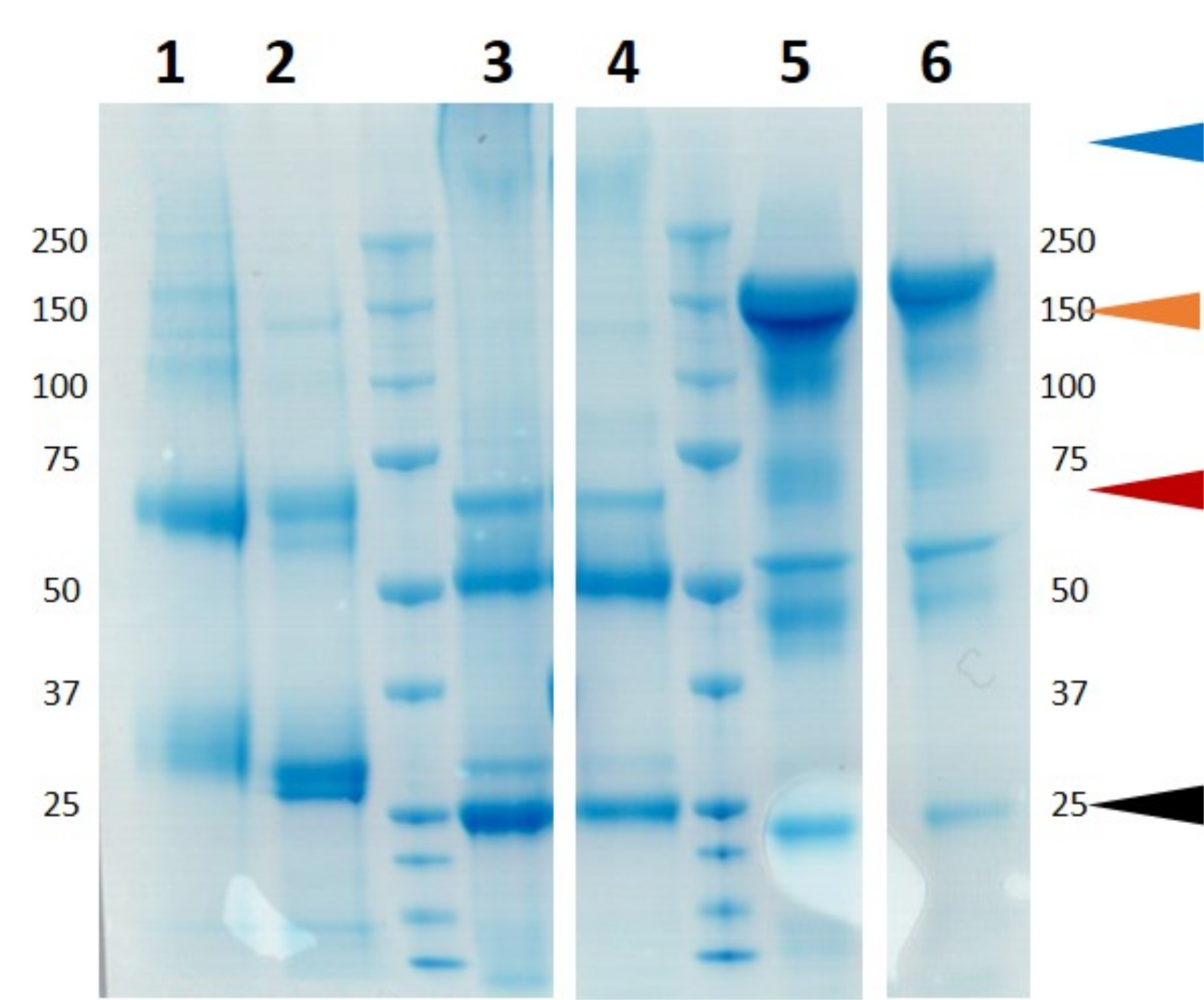

Supplement: Supplementary file 1 — Figure S1 SDS‐PAGE of purified murine and human PIGS (without antigen) produced from CHO cells, under non‐reducing and reducing conditions. [file PBI-16-1983-s007.png]

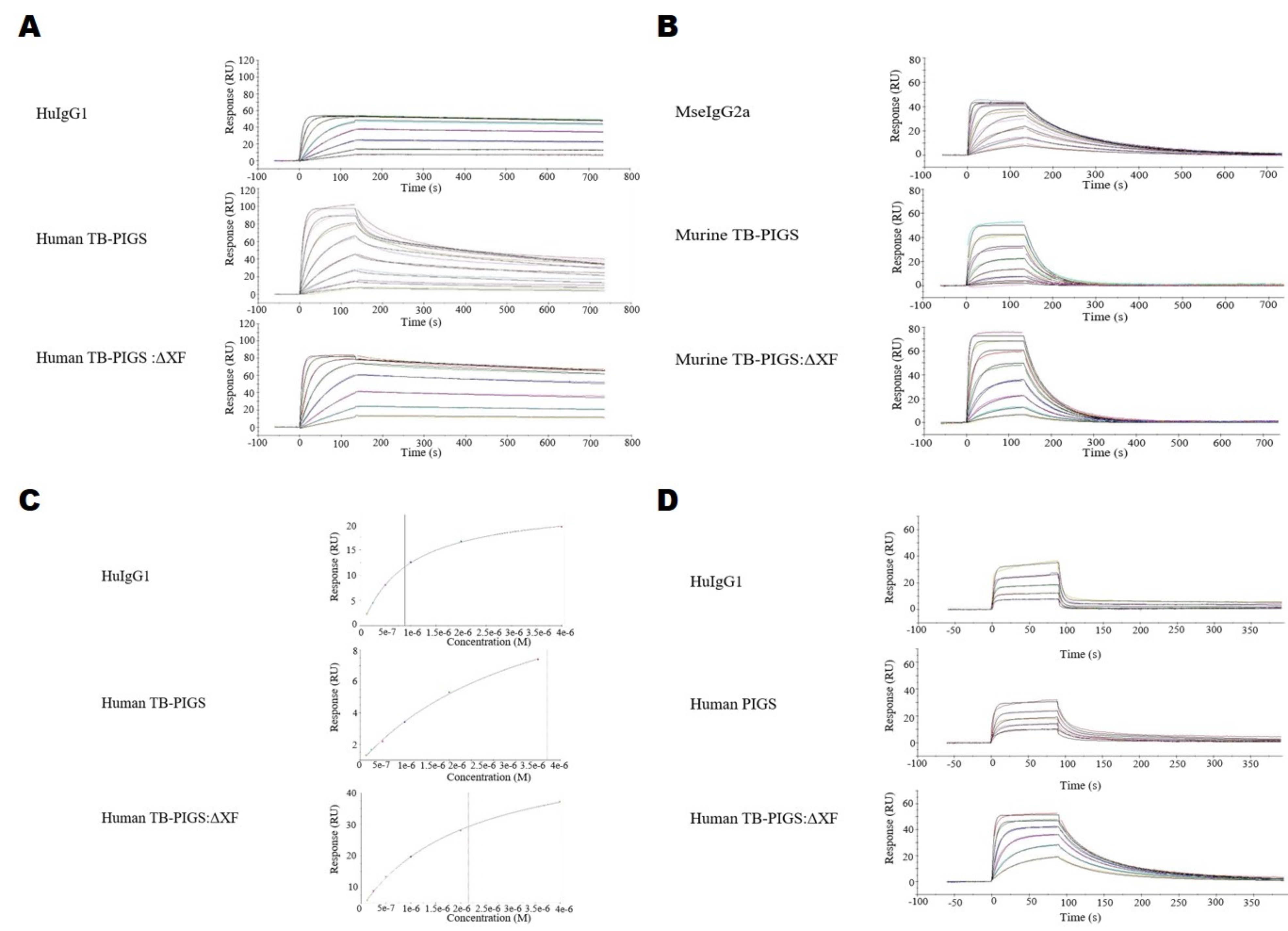

Supplement: Supplementary file 2 — Figure S2 SPR kinetics and affinity analysis of TB‐PIGS binding to FcγRs. [file PBI-16-1983-s006.png]

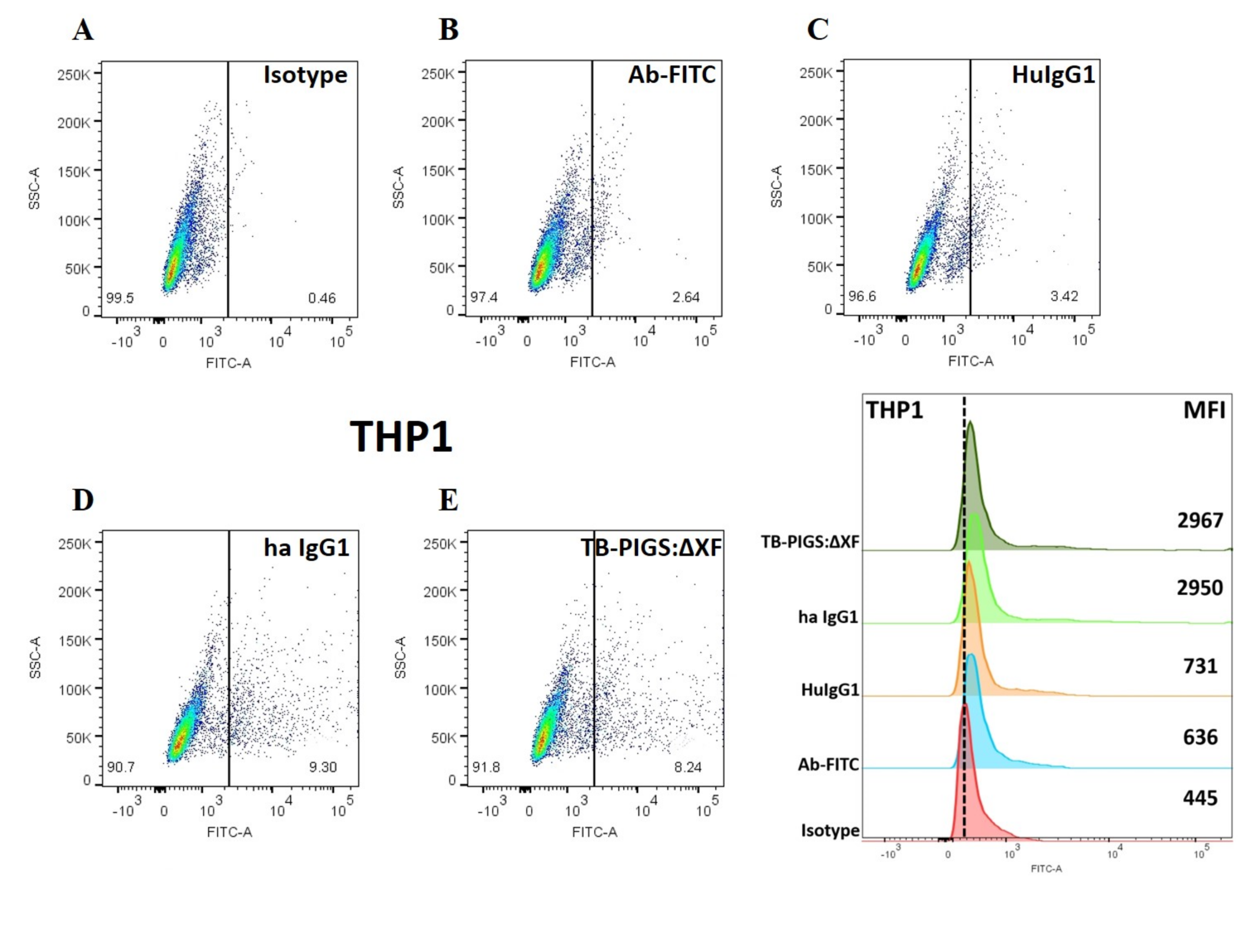

Supplement: Supplementary file 3 — Figure S3 Flow cytometric analysis of human TB‐PIGS:ΔXF with THP1 cells. [file PBI-16-1983-s005.png]

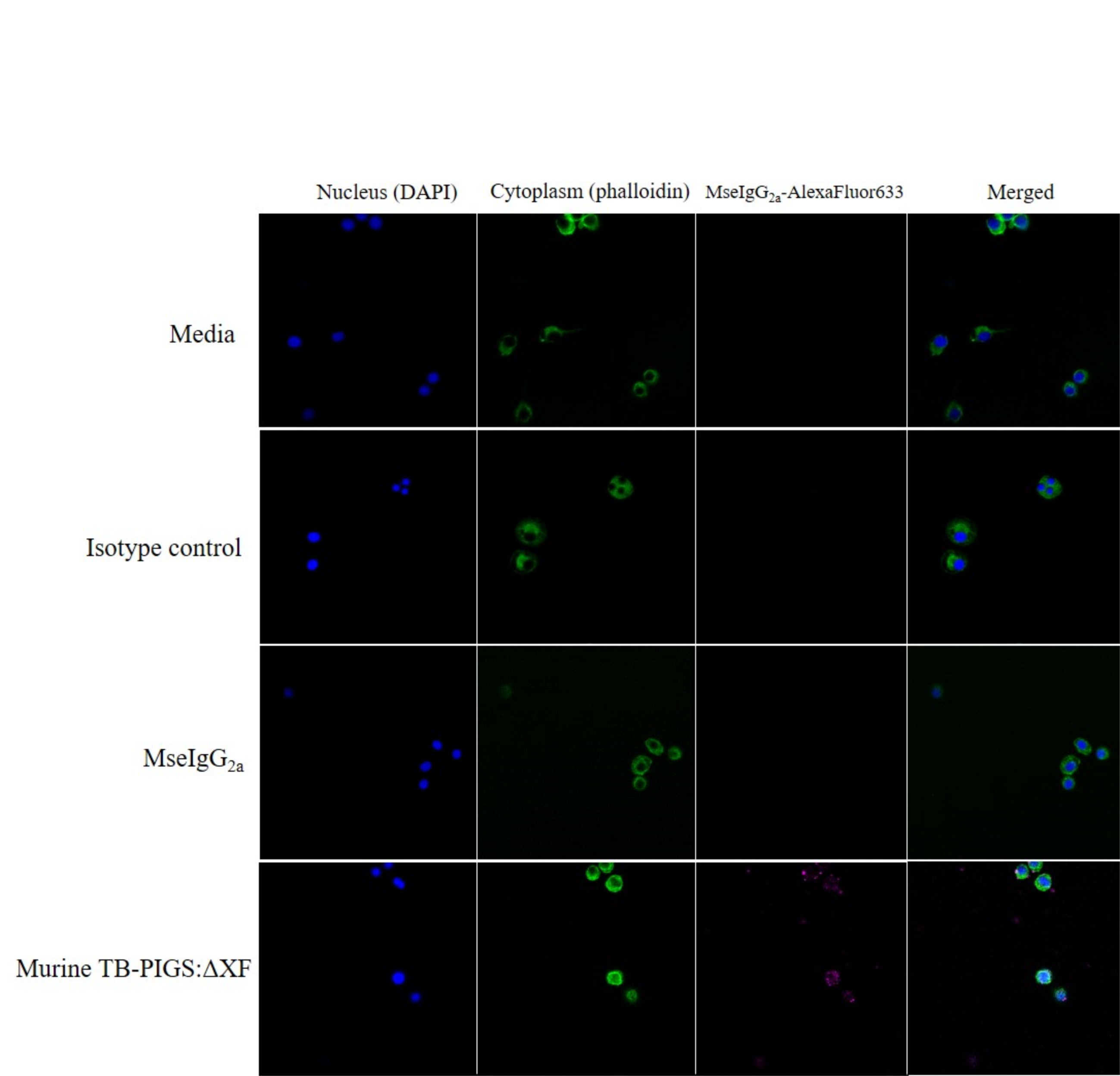

Supplement: Supplementary file 4 — Figure S4 Confocal microscopy of J774 cells with murine TB‐PIGS:ΔXF. [file PBI-16-1983-s001.png]

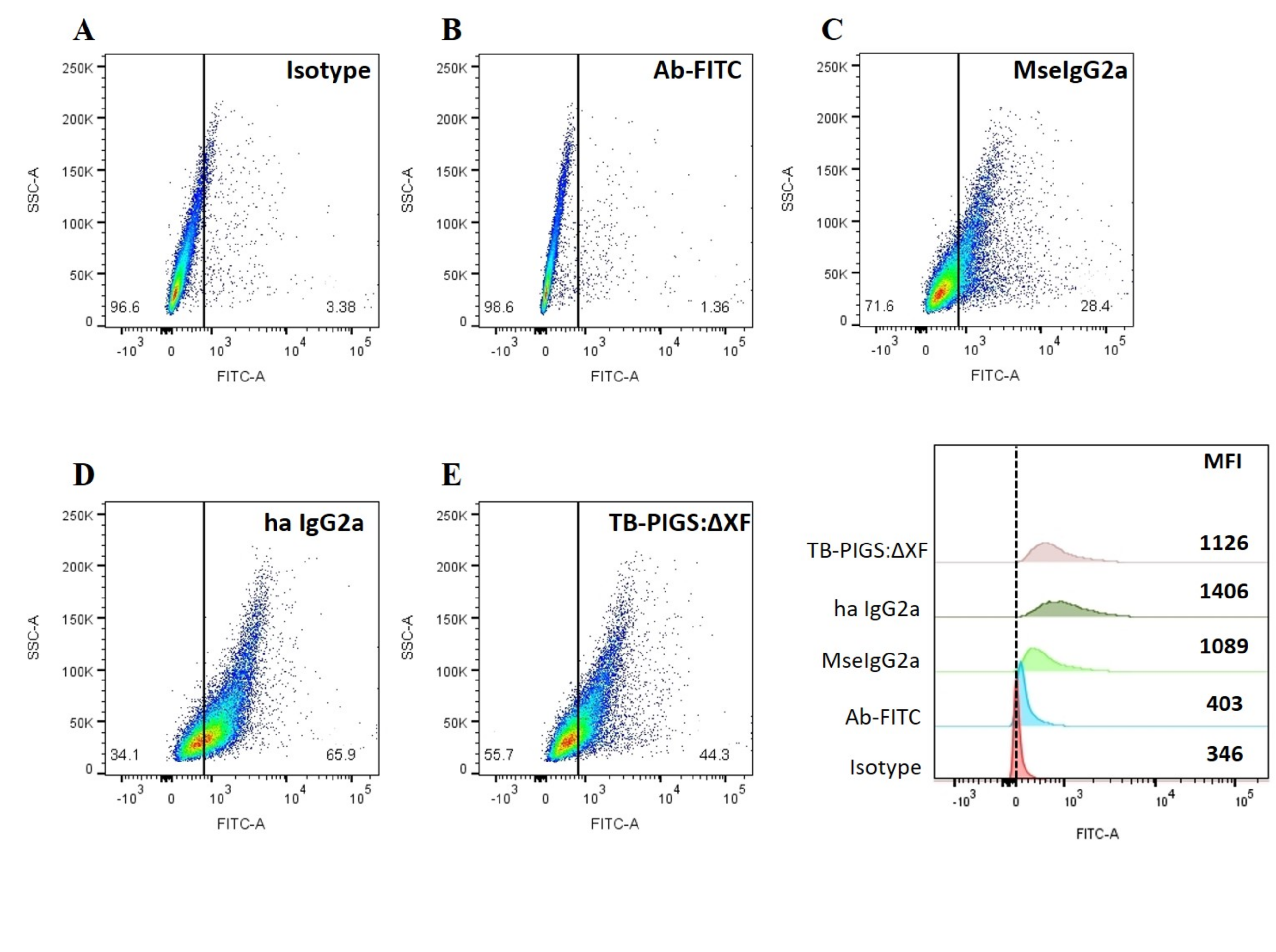

Supplement: Supplementary file 5 — Figure S5 Flow cytometric analysis of murine TB‐PIGS:ΔXF with J774 cells. [file PBI-16-1983-s002.png]
