# Supplementary material for: A polymeric immunoglobulin—antigen fusion protein strategy for enhancing vaccine immunogenicity
Source: Plant Biotechnol J. 2018 Jul 21;16(12):1983–96. doi: 10.1111/pbi.12932 (PMC6230950; doi:10.1111/pbi.12932)
Supplement: Supplementary file 6 — Figure S6 Frequency of antigen‐specific cytokine producing cells after immunisation in transgenic CD64 mice. [file PBI-16-1983-s003.pptx]

## Slide 1
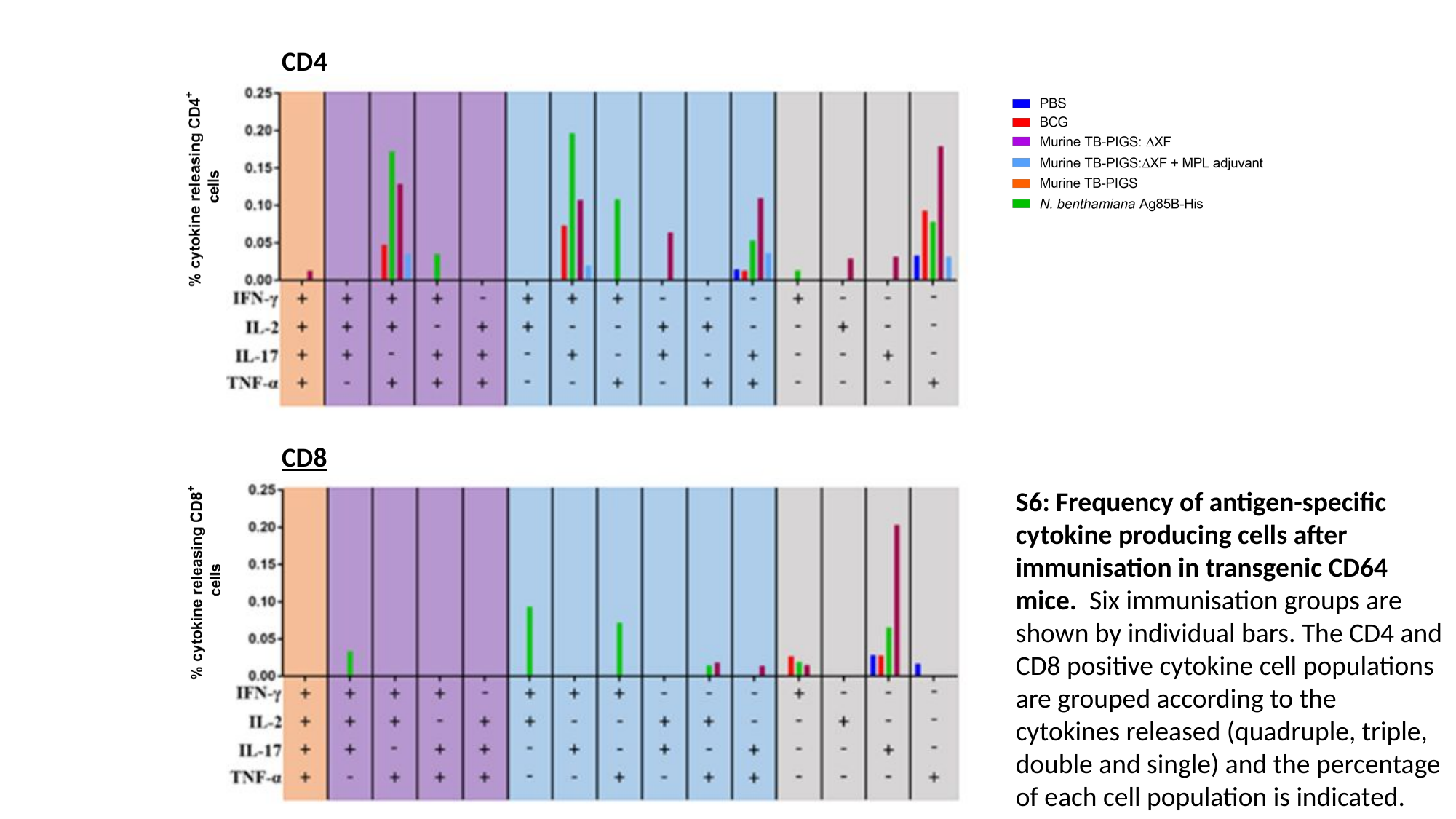

CD4
CD8
CD8
S6: Frequency of antigen-specific cytokine producing cells after immunisation in transgenic CD64 mice. Six immunisation groups are shown by individual bars. The CD4 and CD8 positive cytokine cell populations are grouped according to the cytokines released (quadruple, triple, double and single) and the percentage of each cell population is indicated.
