# Supplementary material for: A polymeric immunoglobulin—antigen fusion protein strategy for enhancing vaccine immunogenicity
Source: Plant Biotechnol J. 2018 Jul 21;16(12):1983–96. doi: 10.1111/pbi.12932 (PMC6230950; doi:10.1111/pbi.12932)
Supplement: Supplementary file 7 — Table S1 Yields for TB‐PIGS produced in plants in mg of protein per kg of leaf mass. Table S2 Kinetic and Affinity values of TB‐PIGS with Fc?Rs. [file PBI-16-1983-s004.docx]

**Supplementary Experimental Procedures**

**Cloning of Ag85B as control protein for expression in *N. benthamiana***

Primer pairs containing BsaI restriction sites and His-tag: 5’-atccatgggcttctctcgtcctggtttgcct- 3’ and 5’ -aggtctagactagtgatgatgatgatgatggccacctgctcccaaagaagattgaag- 3’ were designed to amplify *N-*terminally truncated Ag85B (to remove signal peptide) DNA fragment from pGEM::Ag85B-ESAT6 construct (Dedieu et al. 2010) as well as encoding a His-tag for purification purposes. BsaI restriction digest and T4 DNA ligase (New England Biolabs) was used to insert the desired sequence into the plant expression vector, pTRAk.2.

**Gateway Cloning of TB-PIGS into mammalian expression vector**

AttB1 and attB2 gateway cloning sites were used to clone Ag85B-human PIGS^H^ and Ag85B-murine PIGS^H^ into pDONR-Zeo and the resultant attL1 and attL2 gateway cloning sites were used to clone the desired sequences into destination vector pEF-DEST51 (for CHO cell expression), using the protocols provided by the enzyme manufacturer (Invitrogen).

**Western Blotting to determine structure of PIGS**

Western blotting was performed using nitrocellulose membranes (G E Healthcare) and a semi-dry transfer method (BioRad). The transfer was performed at 0.4 mA/cm^2^ and 50 V for 1 hour 15 minutes. The membrane was incubated in blocking buffer (TBS + 5% non-fat dried skimmed milk (Marvel) + 0.1% Tween20 (SIGMA)) for 1 hour, washed in TBS + 0.1% Tween20, and then incubated in blocking buffer containing antibody for 1 hr. The membrane was washed and developed using ECL Plus Western Blotting detection system (GE Healthcare) and Syngene G:BOX Chemi XT4 system. If secondary antibody was required, the membrane was washed and then incubated in blocking buffer containing antibody for an hour prior to detection. Antibodies used were polyclonal goat α-Mse IgGγ_2a_-HRP (Sigma), goat α-Hu IgGγ_1_-HRP (The Binding Site), and rabbit α-Ag85B sera (Abcam) followed by α-rabbit Ig-HRP antiserum (Sigma).

**Expression and detection of PIGS in CHO cells**

CHO-K1 cells (ATCC) were transfected by either electroporation with plasmids or using FuGENE 6 Transfection Reagent (Promega), using the manufacturer’s instructions. Cells were grown in DMEM medium supplemented with 10% fetal bovine serum, 2 mM *L*-glutamine and 100 IU/ml penicillin and streptomycin at 37ºC, 5% CO_2_ and 90% humidity. Stable transfectants were selected in medium containing 10 μg/ml blasticidin. Clones secreting TB-PIGS were detected by Western blotting using α-Mse IgGγ_2a_ (STAR: 133P) or α-Hu IgGγ_1_ (The Binding Site: AP004) antibodies conjugated to horseradish peroxidase (HRP).

**Cell lines**

**THP-1:** THP-1 cells (ATCC) were grown in RPMI-1640 Medium (SIGMA) with 10% FBS (SIGMA), 2 mM *L*-glutamine (SIGMA) and 100 IU/ml penicillin and streptomycin (SIGMA). Cell density was maintained between 1 x 10^5^ and 2 x 10^6^ viable cells/ml. Cultures flasks were incubated horizontally, at 37°C with 5% CO_2_ and 90% humidity.

**J774:** J774 cells (ATCC) were grown in DMEM (SIGMA) with 10% FBS (SIGMA), 2 mM *L*-glutamine (SIGMA) and 100 IU/ml penicillin and streptomycin (SIGMA). When cells were between 70-90% confluent, they were washed with Dulbecco’s PBS (SIGMA) and then cell dissociation buffer (ThermoFisher Scientific [Gibco]) was added for 5 minutes. Cells were centrifuged at 400g for 5 minutes and re-suspended in fresh medium with an overall 1:6 sub-cultivation dilution. Culture flasks were incubated horizontally, at 37°C with 5% CO_2_ and 90% humidity.

**Flow Cytometry**

If Human FcR block (BD Pharmingen) was utilised, cells were incubated with 2.5 µg per 1 x 10^6^ cells for 20 minutes at room temperature before addition of 25 µg of TB-PIGS or control protein (HuIgG1 or MseIgG2a). Cells were incubated on ice for 2 hours, centrifuged at 300g for 5 minutes and supernatant discarded. Cells were washed with FACS buffer (PBS + 0.5% BSA + 0.1% sodium azide) before addition of fluorophore conjugated antibodies (FITC α-Hu IgG Fc; isotype FITC Mse IgG2a κ; FITC α-Mse IgG2a; isotype FITC Rat IgG2a κ (Biolegend)). Cells were incubated in the dark, on ice, for 20 minutes. Cells were washed with FACS buffer and then re-suspended in 300 µl Cytofix buffer (BD Pharmingen) prior to acquisition on the LSRII machine (BD). Analysis performed using FlowJo version 10.

**Supplementary Figures**

SDS-PAGE of CHO cell expressed protein indicated single chain (black arrow), monomer (red arrow), dimer (orange arrow) and potential polymeric structures (blue arrow) for both murine and human PIGS proteins. Protein bands ran slightly differently to plant produced controls (Lanes 1-2), presumably due to differences in glycosylation.


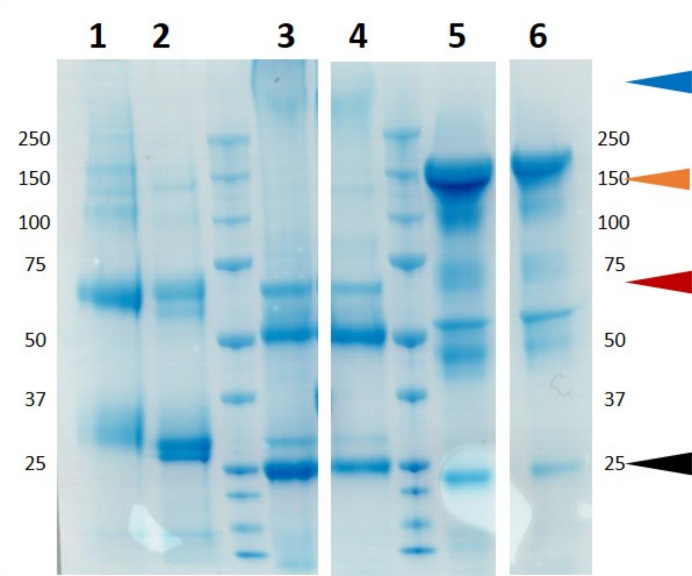


**S1: SDS-PAGE of purified murine and human PIGS (without antigen) produced from CHO cells, under non-reducing and reducing conditions.** A 4-12% Bis-Tris gel in MOPS buffer was used and ~20 μg of protein was loaded in all cases. Lane 1 was *N. benthamiana* produced human PIGS, lane 2 was *N. benthamiana* produced murine PIGS, lane 3 was CHO cell produced human PIGS under non-reducing conditions, lane 4 was CHO cell produced murine PIGS under non-reducing conditions, lane 5 was CHO cell produced human PIGS under reducing conditions and lane 6 was CHO cell produced murine PIGS under reducing conditions.

**
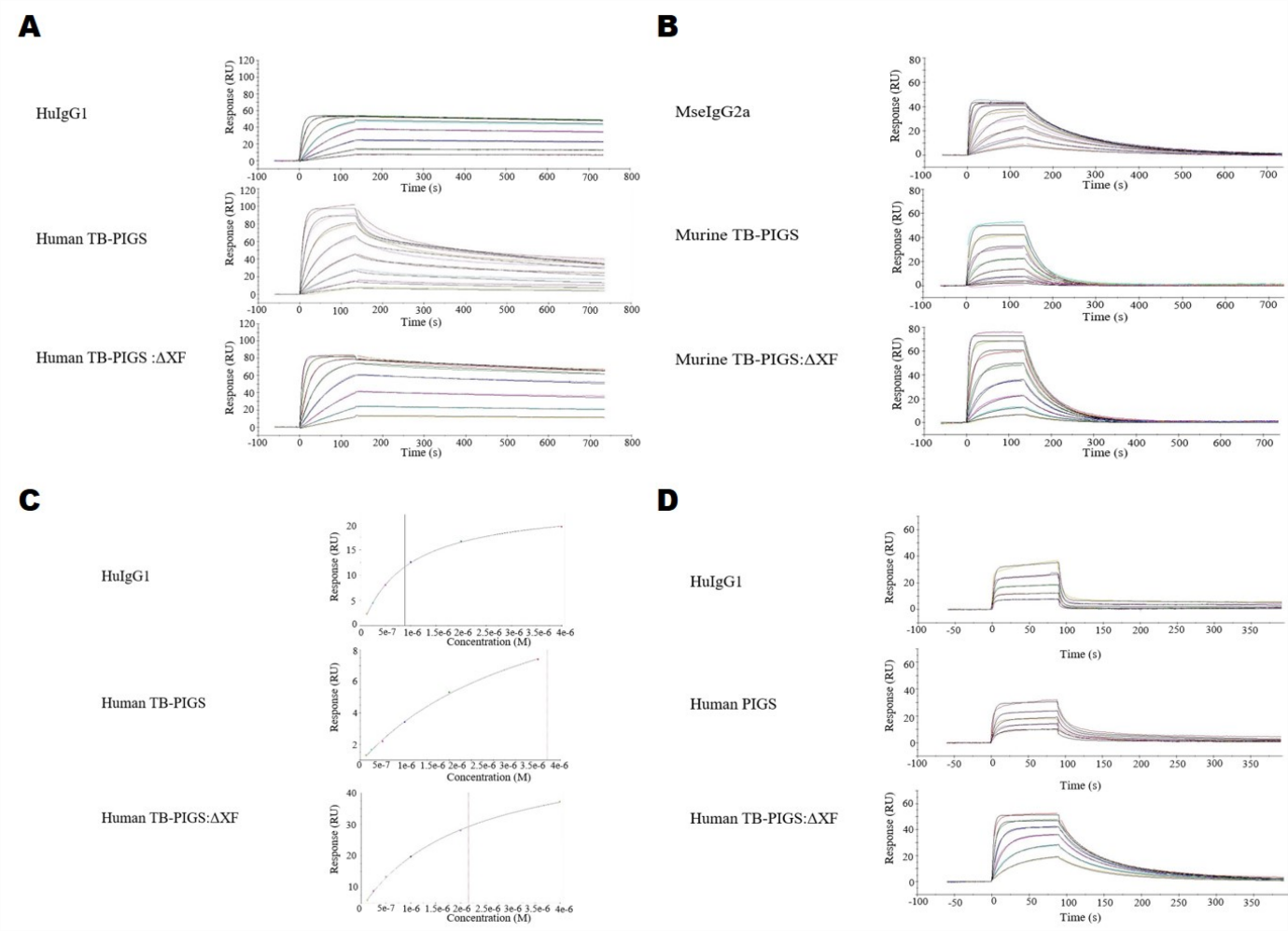
S2: SPR kinetics and affinity analysis of TB-PIGS binding to FcγRs. (A) Human TB-PIGS with human CD64.** Eight different concentrations (240 nM down to 1.875 nM, 1:2 dilutions) of CD64 were passed over the protein A-captured antibodies. 1:1 binding interaction model (black lines) was used for HuIgG1 and human TB-PIGS:ΔXF, whereas ‘Heterogeneous antibody with aglycosylation’ model^*1^ (black lines) was used for wild-type *N. benthamiana* produced human TB-PIGS and the curves were fitted to the experimental data (coloured lines). **(B) Murine TB-PIGS with mouse CD64.** Eight different concentrations (240 nM down to 1.875 nM, 1:2 dilutions) of CD64 were passed over the protein A-captured antibodies/PIGS. 1:1 binding interaction model (black lines) was used for all samples and the curves were fitted to the experimental data (coloured lines). **(C) Steady-state analysis of human TB-PIGS with human CD32a.** Six different concentrations (4000 nM to 125 nM in 2-fold dilutions) of CD32a were passed over the protein A-captured antibodies/PIGS. Affinity values were derived from steady states (using steady-state affinity analysis available in the evaluation software)*.* **(D) Human TB-PIGS with human CD16a.** Five-six different concentrations of CD16a were passed over the protein A-captured antibodies/PIGS. Two-state binding interaction model (black lines) was used for all samples and the curves were fitted to the experimental data (coloured lines).

^*1^This model was created by Stelter *et al.* (manuscript in preparation) and was created based on heterogeneous ligand model, with calculates two separate sets of kinetic and affinity values. One set is defined and corresponds to kinetic/affinity values for aglycosylated heavy chains (as determined for a plant-produced aglycosylated human IgG1) and the second set calculates kinetic/affinity values for the glycosylated portion.

**
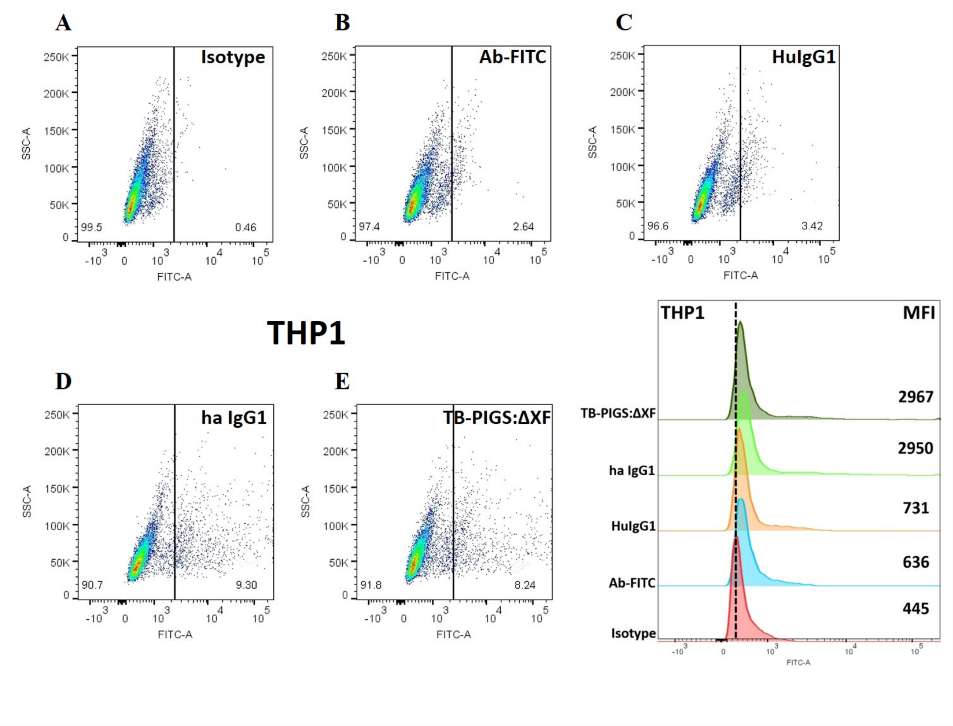
**

**S3: Flow cytometric analysis of human TB-PIGS:ΔXF with THP1 cells.** This data is representative of two repeats. Pseudocolour plots for each sample are shown with percentage values for ‘negative’ population (left of each plot) and percentage values for ‘positive’ FITC population (right of each plot). Half-offset histogram for FITC fluorescence for each sample with their corresponding mean fluorescence intensity (MFI) values given on the right. THP1 cells were incubated with **(A)** isotype FITC labelled MseIgG2a; **(B)** FITC labelled α-HuIgGγ_1_; **(C)** HuIgG1 and then FITC labelled α-HuIgGγ_1_; **(D)** heat aggregated HuIgG1 and then FITC labelled α-HuIgGγ_1_; **(E)** human TB-PIGS:ΔXF and then FITC labelled α-HuIgGγ_1_.

**
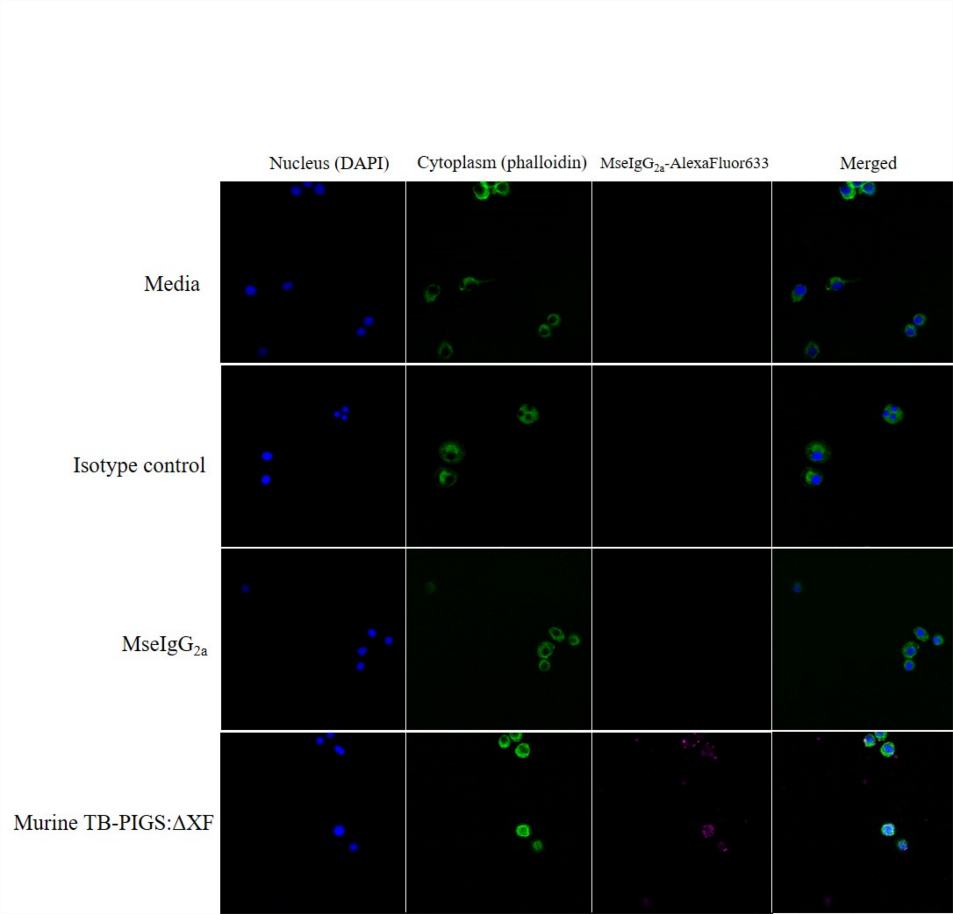
**

**S4: Confocal microscopy of J774 cells with murine TB-PIGS:ΔXF.** Nuclei were stained with DAPI (blue), actin filaments/cytoplasm stained with phalloidin (green; left), MseIgGγ_2a_ stained with AlexaFluor633 (pink; right) and merged images (far right). In descending order, samples incubated with cells were; media only, secondary antibody only, MseIgG2a and murine TB-PIGS:ΔXF.

**
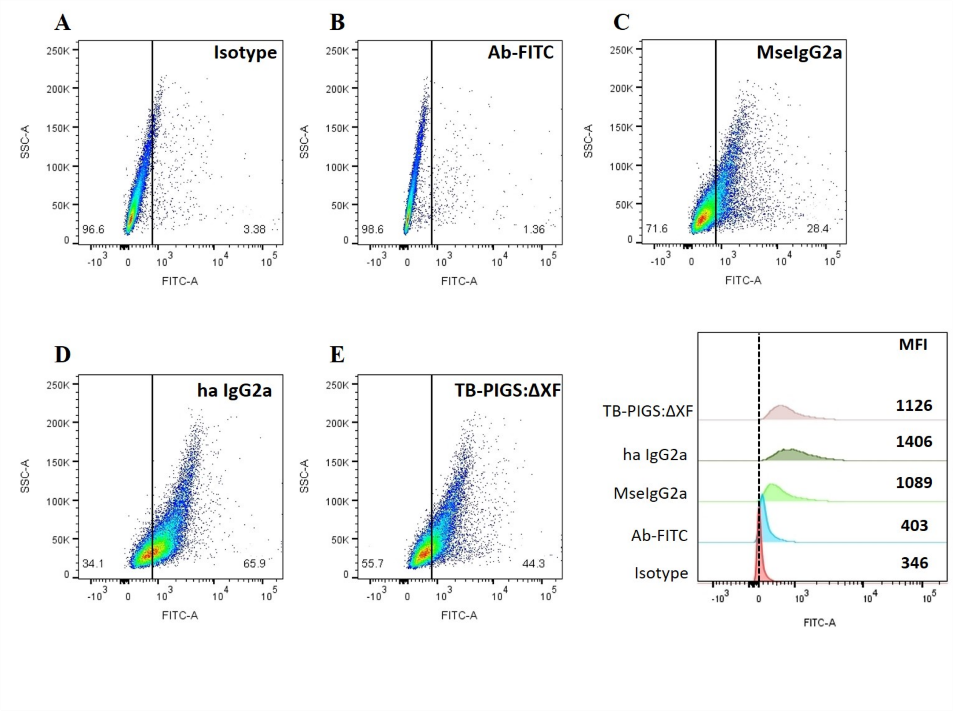
**

**S5: Flow cytometric analysis of murine TB-PIGS:ΔXF with J774 cells.** This data is representative of two repeats. Pseudocolour plots for each sample are shown with percentage values for ‘negative’ population (left of each plot) and percentage values for ‘positive’ FITC population (right of each plot). Half-offset histogram for FITC fluorescence for each sample with their corresponding mean fluorescence intensity (MFI) values given on the right. J774 cells were incubated with **(A)** isotype FITC labelled rat IgG1; **(B)** FITC labelled α-MseIgGγ_2a_; **(C)** MseIgG2a and then FITC labelled α-MseIgGγ_2a_; **(D)** heat aggregated MseIgG2a and then FITC labelled α-MseIgGγ_2a_; **(E)** murine TB-PIGS:ΔXF and then FITC labelled α-MseIgGγ_2a_.

**Table S1: Yields for TB-PIGS produced in plants in mg of protein per kg of leaf mass.** The yield of polymers, dimers and monomers was calculated based on the total yield of protein (mg/kg) and the percentages of each species calculated by native PAGE (NP) or size exclusion chromatography (SEC).

| Construct | Yield (mg/kg) |  | Polymer Yield (mg/kg) | Dimer Yield (mg/kg) | Monomer Yield (mg/kg) |
| --- | --- | --- | --- | --- | --- |
| Murine TB-PIGS | 0.45-1.30 | NP | 0.05-0.13 | 0.14-0.42 | 0.26-0.75 |
| Murine TB-PIGS:ΔXF | 11-33 | NP | 1.87-5.61 | 3.96-11.88 | 5.17-15.51 |
|  |  | SEC | 0 | 4.62-13.86 | - |
| Human TB-PIGS | 4.8-7.1 | NP | 1.73-2.56 | 2.50-3.69 | 0.53-0.78 |
| Human TB-PIGS:ΔXF | 15-46 | NP | 8.85-27.14 | 5.85-17.94 | 0.23-0.69 |
|  |  | SEC | 2.55-9.66 | 4.05-12.42 | 2.40-8.28 |

**Table S2: Kinetic and Affinity values of TB-PIGS with FcγRs.** Two sets of values were generated for wild-type plant produced TB-PIGS with CD64 – one for aglycosylated TB-PIGS and one for glycosylated TB-PIGS present within the sample.

| Kinetic and Affinity of human TB-PIGS with human CD64 | | | | | | | | | | | |
| --- | --- | --- | --- | --- | --- | --- | --- | --- | --- | --- | --- |
| Constructs | **Affinity, K_D_ (nM)** | | **k_on_**  **(10^5^ M^-1^s^-1^)** | | **k_off_**  **(10^-4^ s^-1^)** | | **R_max_**  **(RU)** | | **Chi^2^**  **(RU^2^)** | **Chi^2^/R_max_**  **(%RU)** |  |
| HuIgG1 | 0.27 | | 6.13 | | 1.66 | | 54 | | 0.302 | 0.56 |  |
| Human TB-PIGS | 2.14 | 70.8 | 5.39 | 7.71 | 11.5 | 546 | 67 | 37 | 6.52 | 6.27 |  |
| Human TB-PIGS:ΔXF | 0.41 | | 7.46 | | 3.06 | | 79 | | 0.817 | 1.03 |  |
| Kinetic and Affinity of murine TB-PIGS with mouse CD64 | | | | | | | | | | | |
| MseIgG2a | 4.8 | | 21.8 | | 105 | | 44 | | 0.830 | 1.89 |  |
| Murine TB-PIGS | 50.0 | | 5.0 | | 248 | | 60 | | 1.21 | 2.02 |  |
| Murine TB-PIGS:ΔXF | 16.8 | | 13.8 | | 231 | | 78 | | 1.000 | 1.28 |  |
| Kinetic and Affinity of human TB-PIGS with human CD16a | | | | | | | | | | | |
|  | **Affinity, K_D_ (nM)** | | **k_a1_**  **(10^5^ M^-1^s^-1^)** | **k_d1_**  **(s^-1^)** | **K_a2_**  **(10^-3^s^-1^)** | **k_d2_**  **(10^-3^s^-1^)** | **R_max_**  **(RU)** | | **Chi^2^**  **(RU^2^)** | **Chi^2^/R_max_**  **(%RU)** |  |
| HuIgG1 | 673 | | 0.86 | 0.23 | 2.69 | 0.89 | 72 | | 0.58 | 0.81 |  |
| Human TB-PIGS | 279 | | 1.27 | 0.07 | 4.10 | 3.85 | 31 | | 0.79 | 2.55 |  |
| Human TB-PIGS:ΔXF | 19.7 | | 9.01 | 0.02 | 2.68 | 8.21 | 48 | | 0.29 | 0.60 |  |
| Steady-state analysis of human TB-PIGS with human CD32a | | | | | | | | | | |  |
|  | **Affinity, K_D_ (µM)** | |  |  |  |  | **R_max_**  **(RU)** | | **Chi^2^**  **(RU^2^)** | **Chi^2^/R_max_**  **(%RU)** |  |
| HuIgG1 | 0.88 | |  |  |  |  | 25 | | 0.04 | 0.16 |  |
| Human TB-PIGS | 4.21 | |  |  |  |  | 13 | | 0.01 | 0.08 |  |
| Human TB-PIGS:ΔXF | 2.16 | |  |  |  |  | 52 | | 0.08 | 0.15 |  |
